# Tumor immunological phenotype-derived gene classification predicts prognosis, treatment response, and drug candidates in ovarian cancer

**DOI:** 10.1016/j.gendis.2023.101173

**Published:** 2023-11-21

**Authors:** Chengbin Guo, Yuqin Tang, Zhihai Liu, Chuanliang Chen, Xun Hu, Yongqiang Zhang

**Affiliations:** a Guangzhou Women and Children's Medical Center, Guangzhou Medical University, Guangzhou, Guangdong 510623, China; b Clinical Bioinformatics Experimental Center, Henan Provincial People's Hospital, People's Hospital of Zhengzhou University, Zhengzhou, Henan 450003, China; c School of Pharmacy, Macau University of Science and Technology, Taipa, Macau 999078, China; d Clinical Research Center, The Second Affiliated Hospital, School of Medicine, Zhejiang University, Hangzhou, Zhejiang 310003, China; e Biorepository, State Key Laboratory of Biotherapy, West China Hospital, Sichuan University, Chengdu, Sichuan 610041, China; f West China School of Medicine, West China Hospital, Sichuan University, Chengdu, Sichuan 610041, China

The high recurrence and low responsiveness to immunotherapy make ovarian cancer (OC) the most lethal gynecological malignancy. Tumor microenvironment is critical in risk stratification and the discovery of molecular targets. We developed a prognostic classification for OC, which could also predict the prognosis of other gynecological cancers including breast cancer, endometrial cancer, and cervical cancer. Somatic mutation, hallmark pathways, and immune landscapes were characterized. Integrative analysis of immune checkpoints and multiple immune signatures revealed the low-risk group responds better to immune checkpoint inhibitors, which was validated by an external immunotherapeutic cohort (IMvigor210). Single-cell RNA sequencing (scRNA-seq) confirmed the high expression of *SERPINB1* and *SERPINB9* in dendritic cells, and AlphaFold2 was used to infer their 3D protein structures. Putative molecular compounds binding to *SERPINB1*/*SERPINB9* were predicted by virtual screening.

“Tumor immunological phenotype (TIP) genes” were proposed to classify tumors into “hot” (inflamed) or “cold” (non-inflamed) status.^1^^,^^2^ We have established a “TIP genes"-related signature in hepatocellular carcinoma,^3^ but the utility in other cancers for prognosis, therapeutic response, and drug discovery remains elusive. In this preliminary study, we found that higher TIP scores could exert better survival in the TCGA-OV dataset (Fig. S1A). Besides, the TIP score was positively correlated with multiple immune-related indicators including CD8/CD4 T cells, the expression of *PD-1*/*CTLA-4*, immune/stromal/estimate score, and cytotoxic T lymphocyte (CTL)/cytolytic activity (CYT)/tumor immune escape/tumor inflammation signature (TIS) score, while negatively correlated with tumor purity (Fig. S1B–D).

Next, we performed weighted gene co-expression network analysis as we previously described,^4^ dividing genes into 14 different modules (Fig. 1A). The MEblue module was identified to be most related to the TIP score (Fig. 1B). Kyoto Encyclopedia of Genes and Genomes enrichment indicated it was mostly involved in pathways of cytokine–cytokine receptor interaction (Fig. 1C). Eighty-nine prognostic genes in the MEblue by UniCox analysis were obtained and inputted into least absolute shrinkage and selection operator cox regression analysis (Table S1). The resulting 32 genes were incorporated into stepwise regression, generating 13 genes (*CXCL9*, *PDP1*, *GTF2F2*, *VSIG4*, *ELP3*, *IL2RG*, *IL2*7RA, *SERPINB9*, *TSPAN6*, *CSPG5*, *PABPN1*, SERPINB1, and *ME1*) to construct tumor immunological phenotype-related gene index (TIPRGPI) (Fig. 1D). All OC patients from the training dataset (TCGA-OV) were categorized into distinct risk groups by the median risk score (Fig. 1E). Both Kaplan-Meire survival analysis and time-dependent receiver operating characteristic (tROC) curves showed excellent performance of TIPRGPI (Fig. 1F, G). Two external validation sets (validation dataset 1: GSE32062; validation dataset 2: a meta-array dataset combining GSE17260, GSE9891, and GSE26193) indicated the comparable results (Fig. S2). Additionally, TIPRGPI had prognostic values for progression-free interval, disease-specific survival, and disease-free interval of OC (Fig. S3). Importantly, TIPRGPI was superior to several existing signatures and popular immune-related biomarkers in 3-year and 5-year overall survival (OS) prediction (Fig. 1H, I), and tROC curves suggested TIPRGPI was the most accurate model among them (Fig. 1J). Moreover, TIPRGPI had the highest hazard ratio (Fig. 1K) or C-index in OS prediction of OC (Fig. 1L). Importantly, univariate and multivariate analyses suggested TIPRGPI was an independent indicator (Fig. S4A). A TIPRGPI-integrated nomogram was established with tumor burden, age, and stage (Fig. S4B). Calibration plots showed its good consistency (Fig. S4C), and tROC curves showed excellent performance (Fig. S4D). Decision curve analysis curves suggested it had better net benefit than clinicopathological indicators (Fig. S4E, F), and Kaplan-Meire analysis further validated its prognostic predictive capability (Fig. S4G). These findings demonstrated that TIPRGPI is a reliable prognostic classifier in OC and may guide personalized survival prediction.

**Figure 1:**
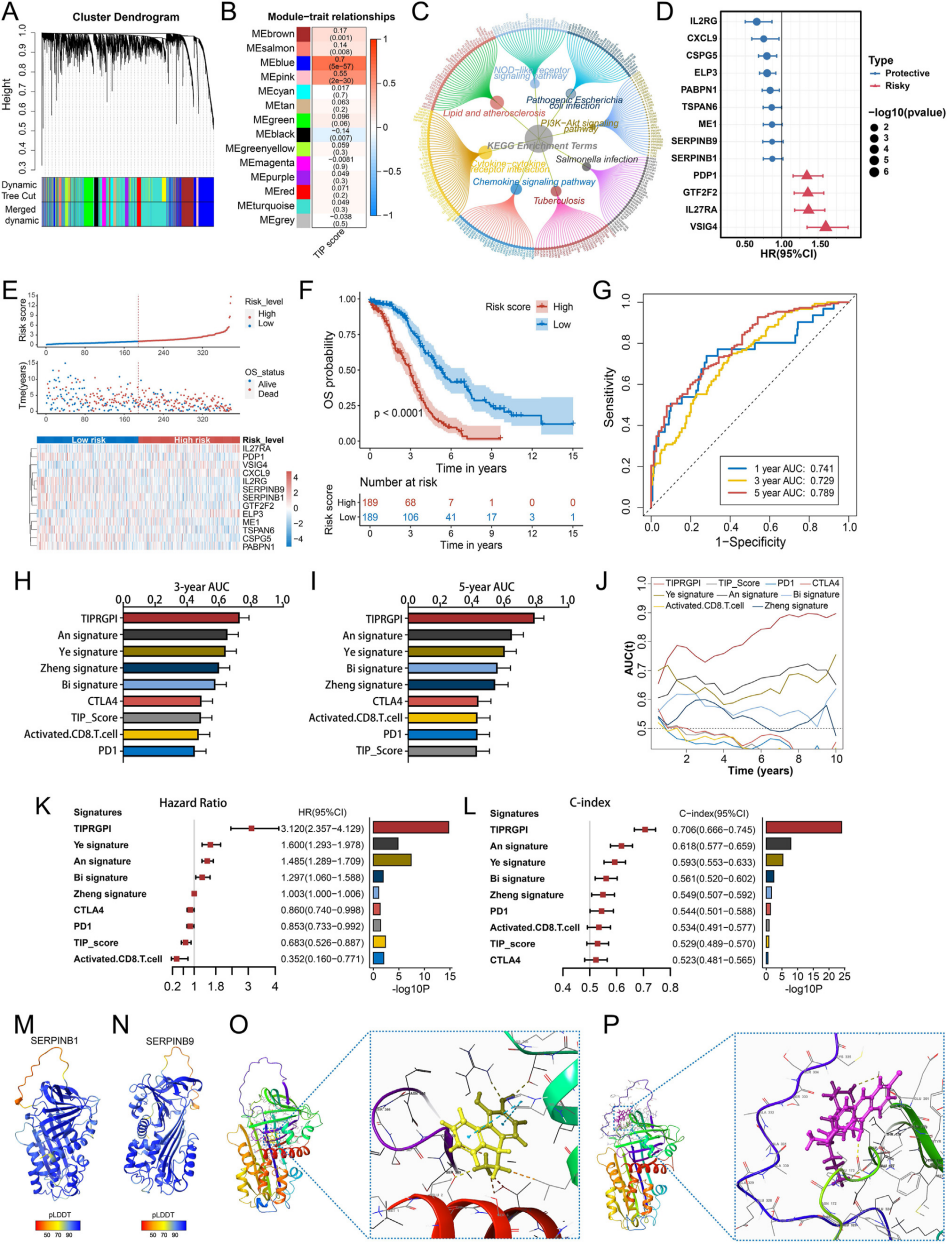
TIP-related gene signature for prediction of survival and drug candidates in OC. **(A)** Cluster dendrogram of genes included in various modules (represented by different colors). **(B)** Correlation analysis of TIP score and module eigengenes. **(C)** Kyoto Encyclopedia of Genes and Genomes (KEGG) enrichment analysis of the blue module genes. **(D)** HR with 95 % confidence interval (CI) and significant level of each gene in the established TIPRGPI signature calculated by MultiCox. **(E)** Risk score distribution, OS status, and the expression levels of the 13 TIPRGPI genes for patients in the low- and high-risk groups from the TCGA-OV (*n* = 378). **(F)** Kaplan–Meier survival analysis of low- and high-risk groups for OS of OC. **(G)** Time-dependent receiver operating characteristic (tROC) curves for the OS of three datasets. **(H, I)** AUC comparisons of the 3-year (H) and 5-year (I) survival in OC for TIPRGPI and other published signatures or immunotherapeutic biomarkers. **(J)** Time-dependent receiver operating characteristic (tROC) curves for TIPRGPI and other signatures or immunotherapeutic biomarkers. **(K)** Comparison of TIPRGPI and other signatures on HR. **(L)** Comparison of TIPRGPI and other signatures on C-index. ^∗∗∗∗^*P* < 0.0001. **(M, N)** 3D protein structures predicted for *SERPINB1* (M) and *SERPINB9* (N) by AF2 (relaxed models), respectively. **(O, P)** The docking diagrams showing the interaction between the potential target drugs and the two protein models. AUC, area under the ROC curve; HR, hazard ratio; OC, ovarian cancer; OS, overall survival; TIP, tumor immunological phenotype.

To evaluate whether TIPRGPI could predict the outcome of other gynecological tumors, all patients of the datasets of cervical cancer, breast cancer, and endometrial cancer from TCGA were categorized into different risk groups by TIPRGPI in the same way. Notably, TIPRGPI could classify them into two groups with distinct outcomes (Fig. S5A–C). The tROC curves showed that TIPRGPI had the potential to predict the OS for these gynecological tumors (Fig. S5D–F), and higher risk scores also indicated worse progression-free interval and disease-specific survival in these cancers (Fig. S5G–L). These results indicated the broad applicability of TIPRGPI in other gynecological tumors.

Moreover, different immune microenvironments were discovered. As shown in Figure S6A, TIPRGPI was negatively correlated with TIP/CTL/CYT/TIS score and immune score, and boxplots verified the differential levels of TIP/CTL/CYT/TIS score between different risk groups (Fig. S6B). The network illustrated four clusters of 30 immune cell types deconvoluted by ssGSEA (Fig. S6C). Six of the seven intersected immune cell types (Fig. S6F) shared by correlation analysis (Fig. S6D) and differential analysis (Fig. S6E) were associated with the OS of OC. Besides, TIPRGPI was significantly correlated with targeted therapy-associated gene signatures (Fig. S6G) and the cancer–immunity cycle (Fig. S6H). It was observed that most immune-related genes had higher levels in the TIPRGPI low-risk group (Fig. S6I). Then, waterfall plots were drawn to show mutation profiles regarding TIPRGPI (Fig. S7A, B). The forest plot exhibited that *KMT2C*, *BRCA1*, and *CSMD1* mutated more frequently in the low-risk group (Fig. S7C), and a lollipop chart was depicted to show the detailed mutation sites of *TP53* (Fig. S7D). The co-occurrences and mutual exclusions of the top 25 mutated genes were also calculated (Fig S7E). Interestingly, tumor mutational burden was negatively associated with TIPRGPI (Fig. S7F, G), and Kaplan–Meier curves for stratified survival analysis by tumor mutational burden and TIPRGPI were plotted (Fig. S7H, I). For hallmark pathways, 11 of them were significantly different between the two groups (Fig. S7J), and oncogenic pathways were confirmed to impose a significant impact on the prognosis of OC (Fig. S7K).

For immunotherapy response prediction, TIPRGPI was negatively correlated with well-known immune checkpoints (*CTLA-4*, *PDL1*, *etc*.) (Fig. S8A, B). Most immune-related metagene signatures were higher in the TIPRGPI low-risk group (Fig. S8C). T cell receptor richness and diversity were significantly lower in the high-risk group (Fig. S8D), and immunophenoscore score was significantly elevated in the low-risk group (Fig. S8E). The IMvigor210 immunotherapeutic cohort was used for further investigation. We started with the confirmation of the prognostic value of TIPRGPI (Fig. S8F), followed by the comparisons of risk scores in stable disease/progressive disease and complete response/partial response groups (Fig. S8G), and differential distribution of stable disease/progressive disease and complete response/partial response patients was observed between distinct risk groups (Fig. S8H). The ROC curve also demonstrated its good performance for immunotherapeutic response prediction (Fig. S8I). Finally, TIPRGPI also showed indicative for chemo-/targeted-therapy by the “pRRophetic” algorithm (Fig. S8J).

Based on the GSE165897 scRNA-seq dataset, three major cell types were preliminarily clustered (Fig. S9A–C). *IL2RG*, *SERPINB1*, and *SERPINB9* were found highly expressed in immune cells. We then extracted immune cells for dimensionality reduction and found *IL2RG* was mainly expressed in T cells while *SERPINB1* and *SERPINB9* were mainly expressed in dendritic cells (Fig. S9D–F). Subsequently, AlphaFold2 was used to predict 3D structures of *SERPINB1*/*SERPINB9* based on their FASTA sequences (Table S2 and Fig. S10A, B). SERPINB1_3 (Fig. 1M) and SERPINB9_3 (Fig. 1N) gained the highest pLDDT score and were used for molecular docking. The top 15 drugs with the highest docking score from the Therapeutic Target Database were shown in Figure S10C, D and Table S3, 4. 9-Aminomethyl-9H-fluorene-3, 4-diol (Fig. 1O) and CGP 40336A (Fig. 1P) were considered the best small molecules for *SERPINB1* and *SERPINB9*, respectively.

Collectively, TIPRGPI could predict the prognosis and therapeutic efficacy in OC, and it also showed indicative value for other gynecological cancers. By employing scRNA-seq analysis, 3D protein structure prediction, and molecular docking, putative drugs were virtually predicted to promote the activities of dendritic cells in the microenvironment of OC. Our study highlighted “TIP genes"-guided strategy for gene classifier and key target identification in pan-cancer and AI-based protein prediction and structure-based screening for drug discovery.

## Author contributions

Y.Z. conceived the study. C.G., Y.T., and Y.Z. contributed to data collection, analysis, and interpretation. Z.L. helped with data visualization. Y.Z. and C.G. completed the drafting of the manuscript. C.C., X.H., and Y.Z. supervised the study. All authors contributed to the article and approved the submitted version.

## Data availability

Publicly available datasets were analyzed in this study. These data can be found at https://portal.gdc.cancer.gov/and https://www.ncbi.nlm.nih.gov/geo/query/acc.cgi.

## Conflict of interests

The authors declare they have no conflict of interests.

## Funding

This work was supported by the 10.13039/501100002858China Postdoctoral Science Foundation (No. 2022M720896).
