# Supplementary material for: Tumor immunological phenotype-derived gene classification predicts prognosis, treatment response, and drug candidates in ovarian cancer
Source: Genes Dis. 2023 Nov 21;11(5):101173. doi: 10.1016/j.gendis.2023.101173 (PMC11176645; doi:10.1016/j.gendis.2023.101173)
Supplement: Multimedia component 1 [file mmc1.docx]

## Tumor immunological phenotype-derived gene classification predicts prognosis, treatment response, and drug candidates in ovarian cancer

## Supplementary Materials and Methods

## Data collection

The bulk RNA-seq profiles of 378 OC patients with corresponding demographics were obtained from the UCSC Xena data portal (<http://xena.ucsc.edu/>) [1]. Clinical outcomes including overall survival (OS), progression-free interval (PFI), disease-free interval (DFI), and disease-specific survival (DSS) were also retrieved. Those patients with incomplete OS information were excluded. Moreover, four transcriptomic microarray datasets containing a total of 707 OC patients were achieved from the Gene Expression Omnibus (GEO) database (https://www.ncbi.nlm.nih.gov/geo/), including GSE32062 (*n* =260, GPL6480), GSE17260 (*n* =110, GPL6480), GSE9891 (*n* = 258, GPL570), and GSE26193 (n =79, GPL570). Data were pre-processed as previously described [2-4]. *Z*-score transformation was used for further normalization and the “sva” package was utilized for batch effect removal [4-7]. The whole TCGA dataset (TCGA-OV) was used as the training set for model construction, the GSE32062 set served as validation set 1 and the other three microarray datasets were merged into validation set 2 (meta-array set) for independent evaluation (Table S5). Additionally, somatic mutation data for OC patients and transcriptional data of other gynecologic cancer, including cervical cancer (TCGA-CESC, *n* = 273), breast cancer (TCGA-BRCA, *n* = 1044), and endometrial cancer (TCGA-UCEC, *n* = 523) were also acquired.

## Calculation of TIP score and its clinical implication and immune correlation in OC

TIP score was computed as described in our previous study [8]. Prognostic associations were analyzed by plotting Kaplan-Meier (K-M) plots for OS, DFI, PFI, and DSS to compare the differences of OC patients in distinct TIP score groups by using the “survminer” package.

Spearman correlation analysis between TIP score and well-known immune signatures was estimated. Among them, the immune/stromal/estimate score and tumor purity were calculated by using the ESTIMATE algorithm [9], and cytotoxic T lymphocyte (CTL) score reflecting the tumor-inﬁltrating CD8+ function was computed by five reported genes [10]. Additionally, the geometric mean of *PRF1* and *GZMA* was used to calculate the cytolytic activity (CYT) score [11-14], and tumor inflammation signature (TIS) was inferred as previously reported [15]. Moreover, we defined tumor immune escape (TIE) score using 31 reported core immune escape-related genes [16] by single-sample gene set enrichment analysis (ssGSEA). Meanwhile, the infiltration of activated CD4 and the activated CD8 cells was deconvoluted by ssGSEA using the “GSVA” package [17, 18].

## Model construction and survival analysis

Weighted gene co-expression network analysis (WGCNA) is a useful tool to identify specific expression patterns from multiple samples. Herein, we performed WGCNA to identify the most related module for the TIP score as we previously described [1], which was completed based on the RNA-seq data of the TCGA-OV set via the “WGCNA” package. The most correlated gene module was then annotated by Gene Ontology (GO) and Kyoto Encyclopedia of Genes and Genomes (KEGG) pathway analysis via the “clusterProfiler” package [19].

To develop a prognostic gene signature, the module was subjected to univariate Cox (UniCox) regression to screen prognostic genes, which were subsequently used to generate the most reliable subset of prognostic genes by using 200 times least absolute shrinkage and selection operator (LASSO) via the “glmnet” package [20]. Akaike information criterion (AIC)-based stepwise Cox regression was further used to develop the TIPRGPI classifier, and the risk score was defined as the sum of the expression of selected genes and the corresponding coefficients.

The TIPRGPI risk scores for the TCGA-OV training set, GSE32062 validation set, and meta-array validation set were calculated, and patients were divided into high- or low-risk groups by the median score of the training set. Kaplan-Meier curves and time-dependent receiver operating characteristic (tROC) plots were depicted to evaluate the performance of TIPRGPI. Additionally, the prognostic significance of TIPRGPI for PFI, DFI, and DSS of OC was also assessed by Kaplan-Meier analysis with log-rank tests. Moreover, we compared the area under the ROC curve (AUC) values, HRs, and C-indexes of TIPRGPI and other prognostic signatures including single immune-related markers such as *PD1*, *CTLA-4*, and TIP score, and several previously reported signatures in OC, ie, an autophagy-related signature by An et al. (“An signature”) [21], a glycolysis-related signature by Bi et al. (“Bi signature”) [22], a ferroptosis-related gene signature by Ye et al. (“Ye signature”) [23], and a tumor microenvironment-related signature by Zheng et al. (“Zheng signature”) [24]. The utility of TIPRGPI for prognostication of other gynecologic cancers was also investigated by Kaplan-Meier curves. Univariate and multivariate analyses were performed to pick out independent indicators of OC. Based on the result of univariate analysis, an integrated nomogram was established by “rms” package, and its performance was assessed by calibration curves [8, 25, 26]. Decision curve analysis (DCA) was further conducted to examine the potential clinical benefits of the nomogram and other characteristics. Besides, Kaplan-Meier and tROC curves were further used to evaluate the predictive strength for clinical survival of the nomogram-derived system.

## Characterization of immune features of TIPRGPI

Based on the TCGA-OV dataset, we examined the Spearman correlations between TIPRGPI and multiple immune signatures, followed by differential analysis. The composition of TIME cells was estimated by ssGSEA using the “GSVA” package [17, 18, 26] with published gene sets for 15 adaptive immune cell types, 13 innate immune cell types, and two stromal components (fibroblasts and endothelial cells) [8, 27, 28]. An interactive network was drawn to show the comprehensive interaction of the TIME cell types. The correlation between TIME cell infiltration and TIPRGPI score was analyzed and differential analysis was conducted. Besides, we comprehensively evaluated the correlations between the TIPRGPI risk score and several previously reported immunotherapeutic signatures and the activities of each step of the cancer immunity cycle the cancer immunity cycle [29-31]. Subsequently, the expression of multiple immunomodulators and other immune-related genes including inflammatory response-related genes, immune escape-related genes, and pyroptosis-related genes was compared between different TIPRGPI risk groups by Wilcoxon test.

## Molecular underpinnings of TIPRGPI

Somatic mutation information of OC samples were acquired and analyzed with the “maftools” package. Waterfall plots were drawn for the top 20 driver genes with the highest frequency in distinct TIPRGPI risk groups. Tumor mutation burden (TMB) was calculated and compared between different TIPRGPI risk groups [8, 32, 33]. Besides, the activities of 50 hallmark pathways (h.all.v7.1.symbols) underlying TIPRGPI were quantiﬁed via the “GSVA” package [18, 34], followed by the differential analysis of them between two TIPRGPI risk groups with a liner model [35].

## Prediction of therapeutic responsiveness

Based on the TCGA-OV dataset, the expression of well-known immune checkpoints including *TIGIT*, *CD27*, *LAG3*, *CD40LG*, *CTLA-4*, *ICOS*, *PDCD1*, *IDO1*, and *PDL1* were contrasted. Given the tight correlations between the immunotherapy and specific gene sets such as IFN-gamma pathway [36] and pyroptosis-related genes [37, 38], 15 metagene signatures were derived from previous publications and their enrichment scores were computed by ssGSEA method [37, 39-43]. The differences of their enrichment scores between distinct groups were determined by the Wilcoxon test. Moreover, we acquired and analyzed the TCR and BCR repertoire indexes including the Shannon diversity index and the richness for OC patients to assess the indicative value of anti-tumor immune response of the TIPRGPI risk score [44]. Meanwhile, we obtained the Immunophenoscore (IPS) from The Cancer Immunome Atlas (TCIA) (https://tcia.at/home) to further verify the immunotherapeutic sensitivities of TIPRGPI. We also downloaded the expression profile and therapeutic information of the IMvigor210 cohort (*n* = 348) which is a urothelial tumor cohort treated by *PD-L1* blockade [39]. All treatment responses of OC patients were assigned to stable disease (SD) / progressive disease (PD) and complete response (CR) / partial response (PR). Besides, the “pRRophetic” package was utilized to estimate the half maximal inhibitory concentration (IC50) values of 138 drugs to explore the predictive ability of TIPRGPI for chemo-/targeted responses.

## Single-cell RNA sequencing (scRNA-Seq) analysis

We download the GSE165897 scRNA-seq dataset from the GEO database and extracted the untreated samples for further analysis. Cells with proportions of mitochondrial gene expression larger than 7.5% were removed and cells with more than 200 and less than 3000 genes expressed were kept for subsequent analysis. Based on the Elbowplot analysis (data not shown), we chose 30 principal components (PCs) and resolution of 0.6 to cluster the cells. 20 PCs and resolution of 0.5 were used to cluster the immune cells. The “SingleR” package was applied to annotate the immune cell types [45]. Seurat 4.1.0 was applied for the scRNA-seq standard analysis [46].

**3D protein structure prediction and screening of small molecular drugs**

AlphaFold v2.0 was utilized to predict the 3D structures of *SERPINB1* and *SERPINB9* [47, 48]. We first downloaded the genetic databases including UniRef90 [49], MGnify [50], BFD [51], Uniclust30 [52], PDB70 [53], and PDB [54] to conduct ALPHAFOLD2. ‘max_template_date’ was set as the ALPHAFOLD2 model did in CASP14: 14 May 2020. ‘Model_preset’ was set to ‘monomer’ as both *SERPINB1* and *SERPINB9* were single-chain proteins. During modeling, ‘run_relax’ was turned on and performed relaxation to optimize the Assisted Model Building and Energy Refinement (AMBER) molecular dynamics of prediction models. By measuring the predicted local distance difference test (pLDDT) scores on a scale of 0 to 100, the model with the highest confidence was selected. Visualization for the predicted structures of *SERPINB1* and *SERPINB9* was performed using ChimeraX [55].

To screen small molecular drugs, structure data for all drugs in SDF format were downloaded from Therapeutic Target Database (TTD) [56]. The docking pockets of the best ALPHAFOLD2-predicted models for *SERPINB1* and *SERPINB9* were predicted by Deepsite module from PlayMolecule website (www.playmolecule.org). Virtual Screening Workflow module in the Schrödinger suite was used to screen the small molecular drugs docking with the predicted protein models from high-throughput virtual screening (HTVS) mode to standard precision (SP) mode to extra precision (XP) mode [57]. After HTVS mode, 10% of drugs were taken to SP mode and 10% of them were taken to XP mode, and after that, 5% of drugs with advanced scores were selected. Ligand Interaction Diagram module in the Schrödinger suite was used to show the ligand-receptor interaction diagrams.

## Statistical analysis

The correlation between two continuous variables was completed using Spearman correlation by the R package “ggplot2”. Kaplan-Meier analysis with the log-rank test was completed using the “survival” package. Univariate and multivariate analyses to test the independent prognostic value of features were performed by the R package “survival”. The optimal cutoff for subgroup separation in survival analysis was determined by the R package “survminer”. Pearson chi-square test was used to examine the statistical significance of the distribution of categorical variables. The R software (version 4.0.2) was applied for all statistical analyses. Unless otherwise specified, *P* < 0.05 was set as statistical significance.

## Supplementary Discussion

The high recurrence rate of OC makes it a devastating malignancy. Increasing evidence reveals that TIME plays a crucial role in the progression and prognosis of ovarian cancer [58, 59]. However, the poor understanding of OC TIME makes it difficult to design personalized therapy strategies, especially for immunotherapy [60]. Thus, more efforts are urgently needed to discover novel biomarkers or risk classifiers regarding the accurate prognostication and therapeutic sensitivity estimation of OC [61-63]. In our previous study, we proposed the utility of an established gene set called “TIP genes” to establish a novel signature for predicting prognosis and immunotherapy efficacy in HCC [8]. This attracted particular interest in its feasibility to identify new clinical models with higher accuracy for OC prognosis prediction, and we even extended the strategy by attempting to screen candidate biomarkers in OC.

We started with multiple statistical and machine learning approaches to establish a 13-gene TIPRGPI model. Survival analysis demonstrated it could stratify OC patients into distinct risk groups for both training and two validation datasets. Univariate and multivariate analysis found TIPRGPI was a superior indicator than other clinical traits for OS prediction, which supported its independent prognostic value.

All 13 genes included in the model were immune-related. *CXCL9*, an important chemokine in OC TIME, the expression level of which was associated with CD8 T cell infiltration in solid tumors and negatively correlated with OC prognosis [64, 65]. Loss of *IL2RG* resulted in severe immunodeficiency in human or animal models [66, 67]. Several studies revealed that *IL27RA* was an immune-related biomarker in TME [68-70]. *SERPINB1*/*9* encoded a member of the serine protease inhibitor family known as serpins, belonging to a subfamily of intracellular serpins, was reported associated with the innate immune system [71, 72].

By comparing TIPRGPI with other existing prognostic signatures and immunotherapeutic indicators in cancer, we found the higher performance of TIPRGPI in multi-item comparisons. Interestingly, TIPRGPI was found to be capable of predicting the prognosis of other gynecological tumors including cervical cancer, breast cancer, and endometrial carcinoma, representing an obvious strength of our study compared to most similar studies that only focused the utility of their models in one cancer type. These results proved that TIPRGPI had a wide range of application value for prognostic studies of gynecological cancers, demonstrating its high reliability and effectiveness. All these findings strongly suggested the feasibility and generality of such a “TIP-genes”-based strategy for prognosis in pan-cancer.

Considering the importance of immune infiltration on the prognosis of OC, we explored the correlation between TIPRGPI risk score and multiple immune-related signatures and found it was negatively associated with TIP/CTL/CYT/TIS score and immune score. Next, correlation and differential analysis of 30 immune cell types showed that patients with high-TIPRGPI risk scores were composed of less protective immune cell infiltration such as activated CD4 T cells and activated CD8 T cells, both of which were considered enhancers of immunotherapeutic response [73, 74], which may partially explain the poorer survival of the high-risk group. The significant associations between TIPRGPI risk score and therapeutic-related signatures and the cancer-immunity cycle, together with the higher expression of multiple immune-related genes in the low-risk group further improved the understanding of the tight linkage between TIPRGPI and immune status. Overall, TIPRGPI was highly correlated with immune infiltration in OC TME and provided novel insights into the relationship between immune infiltration and prognosis.

In the past two decades, immunotherapy against malignant tumors has made unprecedentedly rapid progress. Recently, immune checkpoint inhibitors, including *CTLA-4* and *PD-1/PD-L1* inhibitors, were capable of unleashing the pre-existing immunity against tumor cells [75, 76]. Hence, we tested whether TIPRGPI was useful for the stratification of OC patients by distinct immunotherapy sensitivities and found that the expression levels of most immune checkpoint markers including *CTLA-4* and *PD-1/PD-L1* were higher in the low-TIPRGPI risk group, indicating OC patients with low-risk score might be more sensitive to ICIs. Similarly, TCR repertoire indexes and IPS were significantly higher in the low-risk group, and TIPRGPI also showed great potential for predicting immunotherapy in the IMvigor210 cohort.

To explore the specific cell types in which TIPRGPI genes were mainly expressed, scRNA-seq analysis was conducted. Three main TME cell types were first identified from GSE165897: immune cells, stromal cells, and epithelial cells by recognized markers, respectively [77]. Since *IL2RG*, *SERPINB1*, and *SERPINB9* were mainly expressed in immune cells, immune cells were extracted to identify more specific immune cell types. Then, we found *IL2RG* was mainly expressed in T cells while *SERPINB1* and *SERPINB9* were mainly expressed in dendritic cells (DCs). Thereafter, we performed 3D protein structure prediction of them using AlphaFold2 and utilized the Schrodinger suite to pick out possible small molecules for drug discovery. As a result, 9-Aminomethyl-9H-fluorene-3, 4-diol and CGP 40336A ranked first to form the most stable binding mode for *SERPINB1* and *SERPINB9*, respectively.

# References

1. Zhang Y, Yang Z, Tang Y, Guo C, Lin D, Cheng L*, et al.* Hallmark guided identification and characterization of a novel immune-relevant signature for prognostication of recurrence in stage I–III lung adenocarcinoma. Genes & Diseases 2023, 10(4): 1657-1674.

2. Guo C, Tang Y, Zhang Y, Li G. Mining TCGA Data for Key Biomarkers Related to Immune Microenvironment in Endometrial cancer by Immune Score and Weighted Correlation Network Analysis. Front Mol Biosci 2021, 8: 645388.

3. Tang Y, Zhang Y, Hu X. Identification of Potential Hub Genes Related to Diagnosis and Prognosis of Hepatitis B Virus-Related Hepatocellular Carcinoma via Integrated Bioinformatics Analysis. Biomed Res Int 2020, 2020: 4251761.

4. Wang S, Xiong Y, Zhang Q, Su D, Yu C, Cao Y*, et al.* Clinical significance and immunogenomic landscape analyses of the immune cell signature based prognostic model for patients with breast cancer. Brief Bioinform 2021, 22(4).

5. Leek JT, Johnson WE, Parker HS, Jaffe AE, Storey JD. The sva package for removing batch effects and other unwanted variation in high-throughput experiments. Bioinformatics 2012, 28(6): 882-883.

6. Zhang Y, Tang Y, Guo C, Li G. Integrative analysis identifies key mRNA biomarkers for diagnosis, prognosis, and therapeutic targets of HCV-associated hepatocellular carcinoma. Aging (Albany NY) 2021, 13.

7. Yasrebi H. Comparative study of joint analysis of microarray gene expression data in survival prediction and risk assessment of breast cancer patients. Brief Bioinform 2016, 17(5): 771-785.

8. Tang Y, Guo C, Yang Z, Wang Y, Zhang Y, Wang D. Identification of a Tumor Immunological Phenotype-Related Gene Signature for Predicting Prognosis, Immunotherapy Efficacy, and Drug Candidates in Hepatocellular Carcinoma. Front Immunol 2022, 13: 862527.

9. Yoshihara K, Shahmoradgoli M, Martinez E, Vegesna R, Kim H, Torres-Garcia W*, et al.* Inferring tumour purity and stromal and immune cell admixture from expression data. Nat Commun 2013, 4: 2612.

10. Liu Y, Liang G, Xu H, Dong W, Dong Z, Qiu Z*, et al.* Tumors exploit FTO-mediated regulation of glycolytic metabolism to evade immune surveillance. Cell Metab 2021, 33(6): 1221-1233 e1211.

11. Balli D, Rech AJ, Stanger BZ, Vonderheide RH. Immune Cytolytic Activity Stratifies Molecular Subsets of Human Pancreatic Cancer. Clin Cancer Res 2017, 23(12): 3129-3138.

12. Rooney MS, Shukla SA, Wu CJ, Getz G, Hacohen N. Molecular and genetic properties of tumors associated with local immune cytolytic activity. Cell 2015, 160(1-2): 48-61.

13. Sammut SJ, Crispin-Ortuzar M, Chin SF, Provenzano E, Bardwell HA, Ma W*, et al.* Multi-omic machine learning predictor of breast cancer therapy response. Nature 2022, 601(7894): 623-629.

14. Guo C, Tang Y, Li Q, Yang Z, Guo Y, Chen C*, et al.* Deciphering the immune heterogeneity dominated by natural killer cells with prognostic and therapeutic implications in hepatocellular carcinoma. Comput Biol Med 2023, 158: 106872.

15. Tan L, Qin Y, Xie R, Xia T, Duan X, Peng L*, et al.* N6-methyladenosine-associated prognostic pseudogenes contribute to predicting immunotherapy benefits and therapeutic agents in head and neck squamous cell carcinoma. Theranostics 2022, 12(17): 7267-7288.

16. Wang F, Zheng A, Zhang D, Zou T, Xiao M, Chen J*, et al.* Molecular profiling of core immune-escape genes highlights LCK as an immune-related prognostic biomarker in melanoma. Front Immunol 2022, 13: 1024931.

17. Hanzelmann S, Castelo R, Guinney J. GSVA: gene set variation analysis for microarray and RNA-seq data. BMC Bioinformatics 2013, 14: 7.

18. Subramanian A, Tamayo P, Mootha VK, Mukherjee S, Ebert BL, Gillette MA*, et al.* Gene set enrichment analysis: a knowledge-based approach for interpreting genome-wide expression profiles. Proceedings of the National Academy of Sciences of the United States of America 2005, 102(43): 15545-15550.

19. Yu G, Wang LG, Han Y, He QY. clusterProfiler: an R package for comparing biological themes among gene clusters. OMICS 2012, 16(5): 284-287.

20. Friedman J, Hastie T, Tibshirani R. Regularization Paths for Generalized Linear Models via Coordinate Descent. J Stat Softw 2010, 33(1): 1-22.

21. An Y, Bi F, You Y, Liu X, Yang Q. Development of a Novel Autophagy-related Prognostic Signature for Serous Ovarian Cancer. J Cancer 2018, 9(21): 4058-4071.

22. Bi J, Bi F, Pan X, Yang Q. Establishment of a novel glycolysis-related prognostic gene signature for ovarian cancer and its relationships with immune infiltration of the tumor microenvironment. J Transl Med 2021, 19(1): 382.

23. Ye Y, Dai Q, Li S, He J, Qi H. A Novel Defined Risk Signature of the Ferroptosis-Related Genes for Predicting the Prognosis of Ovarian Cancer. Front Mol Biosci 2021, 8: 645845.

24. Zheng M, Long J, Chelariu-Raicu A, Mullikin H, Vilsmaier T, Vattai A*, et al.* Identification of a Novel Tumor Microenvironment Prognostic Signature for Advanced-Stage Serous Ovarian Cancer. Cancers (Basel) 2021, 13(13).

25. Tang Y, Guo C, Chen C, Zhang Y. Characterization of cellular senescence patterns predicts the prognosis and therapeutic response of hepatocellular carcinoma. Front Mol Biosci 2022, 9: 1100285.

26. Guo C, Tang Y, Yang Z, Li G, Zhang Y. Hallmark-guided subtypes of hepatocellular carcinoma for the identification of immune-related gene classifiers in the prediction of prognosis, treatment efficacy, and drug candidates. Front Immunol 2022, 13: 958161.

27. Charoentong P, Finotello F, Angelova M, Mayer C, Efremova M, Rieder D*, et al.* Pan-cancer Immunogenomic Analyses Reveal Genotype-Immunophenotype Relationships and Predictors of Response to Checkpoint Blockade. Cell reports 2017, 18(1): 248-262.

28. Becht E, Giraldo NA, Lacroix L, Buttard B, Elarouci N, Petitprez F*, et al.* Estimating the population abundance of tissue-infiltrating immune and stromal cell populations using gene expression. Genome Biol 2016, 17(1): 218.

29. Chen DS, Mellman I. Oncology meets immunology: the cancer-immunity cycle. Immunity 2013, 39(1): 1-10.

30. Xu L, Deng C, Pang B, Zhang X, Liu W, Liao G*, et al.* TIP: A Web Server for Resolving Tumor Immunophenotype Profiling. Cancer Res 2018, 78(23): 6575-6580.

31. Hu J, Yu A, Othmane B, Qiu D, Li H, Li C*, et al.* Siglec15 shapes a non-inflamed tumor microenvironment and predicts the molecular subtype in bladder cancer. Theranostics 2021, 11(7): 3089-3108.

32. Sun J, Shi R, Zhang X, Fang D, Rauch J, Lu S*, et al.* Characterization of immune landscape in papillary thyroid cancer reveals distinct tumor immunogenicity and implications for immunotherapy. Oncoimmunology 2021, 10(1): e1964189.

33. Chalmers ZR, Connelly CF, Fabrizio D, Gay L, Ali SM, Ennis R*, et al.* Analysis of 100,000 human cancer genomes reveals the landscape of tumor mutational burden. Genome Med 2017, 9(1): 34.

34. Liberzon A, Birger C, Thorvaldsdottir H, Ghandi M, Mesirov JP, Tamayo P. The Molecular Signatures Database (MSigDB) hallmark gene set collection. Cell Syst 2015, 1(6): 417-425.

35. Lambrechts D, Wauters E, Boeckx B, Aibar S, Nittner D, Burton O*, et al.* Phenotype molding of stromal cells in the lung tumor microenvironment. Nat Med 2018, 24(8): 1277-1289.

36. Xu F, Chen JX, Yang XB, Hong XB, Li ZX, Lin L*, et al.* Analysis of Lung Adenocarcinoma Subtypes Based on Immune Signatures Identifies Clinical Implications for Cancer Therapy. Mol Ther Oncolytics 2020, 17: 241-249.

37. Chen X, Chen H, Yao H, Zhao K, Zhang Y, He D*, et al.* Turning up the heat on non-immunoreactive tumors: pyroptosis influences the tumor immune microenvironment in bladder cancer. Oncogene 2021, 40(45): 6381-6393.

38. Wu J, Zhu Y, Luo M, Li L. Comprehensive Analysis of Pyroptosis-Related Genes and Tumor Microenvironment Infiltration Characterization in Breast Cancer. Front Immunol 2021, 12: 748221.

39. Mariathasan S, Turley SJ, Nickles D, Castiglioni A, Yuen K, Wang Y*, et al.* TGFbeta attenuates tumour response to PD-L1 blockade by contributing to exclusion of T cells. Nature 2018, 554(7693): 544-548.

40. Wang X, Wu S, Liu F, Ke D, Wang X, Pan D*, et al.* An Immunogenic Cell Death-Related Classification Predicts Prognosis and Response to Immunotherapy in Head and Neck Squamous Cell Carcinoma. Front Immunol 2021, 12: 781466.

41. Van Opdenbosch N, Lamkanfi M. Caspases in Cell Death, Inflammation, and Disease. Immunity 2019, 50(6): 1352-1364.

42. Broz P, Pelegrin P, Shao F. The gasdermins, a protein family executing cell death and inflammation. Nat Rev Immunol 2020, 20(3): 143-157.

43. Bergsbaken T, Fink SL, Cookson BT. Pyroptosis: host cell death and inflammation. Nat Rev Microbiol 2009, 7(2): 99-109.

44. Sayaman RW, Saad M, Thorsson V, Hu D, Hendrickx W, Roelands J*, et al.* Germline genetic contribution to the immune landscape of cancer. Immunity 2021, 54(2): 367-386 e368.

45. Aran D, Looney AP, Liu L, Wu E, Fong V, Hsu A*, et al.* Reference-based analysis of lung single-cell sequencing reveals a transitional profibrotic macrophage. Nat Immunol 2019, 20(2): 163-172.

46. Hao Y, Hao S, Andersen-Nissen E, Mauck WM, 3rd, Zheng S, Butler A*, et al.* Integrated analysis of multimodal single-cell data. Cell 2021, 184(13): 3573-3587 e3529.

47. Varadi M, Anyango S, Deshpande M, Nair S, Natassia C, Yordanova G*, et al.* AlphaFold Protein Structure Database: massively expanding the structural coverage of protein-sequence space with high-accuracy models. Nucleic Acids Res 2022, 50(D1): D439-D444.

48. Jumper J, Evans R, Pritzel A, Green T, Figurnov M, Ronneberger O*, et al.* Highly accurate protein structure prediction with AlphaFold. Nature 2021, 596(7873): 583-589.

49. Suzek BE, Huang H, McGarvey P, Mazumder R, Wu CH. UniRef: comprehensive and non-redundant UniProt reference clusters. Bioinformatics 2007, 23(10): 1282-1288.

50. Mitchell AL, Almeida A, Beracochea M, Boland M, Burgin J, Cochrane G*, et al.* MGnify: the microbiome analysis resource in 2020. Nucleic Acids Res 2020, 48(D1): D570-D578.

51. Steinegger M, Mirdita M, Soding J. Protein-level assembly increases protein sequence recovery from metagenomic samples manyfold. Nat Methods 2019, 16(7): 603-606.

52. Mirdita M, von den Driesch L, Galiez C, Martin MJ, Soding J, Steinegger M. Uniclust databases of clustered and deeply annotated protein sequences and alignments. Nucleic Acids Res 2017, 45(D1): D170-D176.

53. Steinegger M, Meier M, Mirdita M, Vohringer H, Haunsberger SJ, Soding J. HH-suite3 for fast remote homology detection and deep protein annotation. BMC Bioinformatics 2019, 20(1): 473.

54. Berman HM, Westbrook J, Feng Z, Gilliland G, Bhat TN, Weissig H*, et al.* The Protein Data Bank. Nucleic Acids Res 2000, 28(1): 235-242.

55. Pettersen EF, Goddard TD, Huang CC, Meng EC, Couch GS, Croll TI*, et al.* UCSF ChimeraX: Structure visualization for researchers, educators, and developers. Protein Sci 2021, 30(1): 70-82.

56. Zhou Y, Zhang Y, Lian X, Li F, Wang C, Zhu F*, et al.* Therapeutic target database update 2022: facilitating drug discovery with enriched comparative data of targeted agents. Nucleic Acids Res 2022, 50(D1): D1398-D1407.

57. Halgren TA, Murphy RB, Friesner RA, Beard HS, Frye LL, Pollard WT*, et al.* Glide: a new approach for rapid, accurate docking and scoring. 2. Enrichment factors in database screening. J Med Chem 2004, 47(7): 1750-1759.

58. Olalekan S, Xie B, Back R, Eckart H, Basu A. Characterizing the tumor microenvironment of metastatic ovarian cancer by single-cell transcriptomics. Cell reports 2021, 35(8): 109165.

59. Schoutrop E, El-Serafi I, Poiret T, Zhao Y, Gultekin O, He R*, et al.* Mesothelin-Specific CAR T Cells Target Ovarian Cancer. Cancer Res 2021, 81(11): 3022-3035.

60. Launonen IM, Lyytikainen N, Casado J, Anttila EA, Szabo A, Haltia UM*, et al.* Single-cell tumor-immune microenvironment of BRCA1/2 mutated high-grade serous ovarian cancer. Nat Commun 2022, 13(1): 835.

61. Moore KN, Pignata S. Trials in progress: IMagyn050/GOG 3015/ENGOT-OV39. A Phase III, multicenter, randomized study of atezolizumab versus placebo administered in combination with paclitaxel, carboplatin, and bevacizumab to patients with newly-diagnosed stage III or stage IV ovarian, fallopian tube, or primary peritoneal cancer. International journal of gynecological cancer : official journal of the International Gynecological Cancer Society 2019.

62. Naumann RW, Coleman RL. Management strategies for recurrent platinum-resistant ovarian cancer. Drugs 2011, 71(11): 1397-1412.

63. Liu YL, Zhou Q, Iasonos A, Emengo VN, Friedman C, Konner JA*, et al.* Subsequent therapies and survival after immunotherapy in recurrent ovarian cancer. Gynecol Oncol 2019, 155(1): 51-57.

64. Millstein J, Budden T, Goode EL, Anglesio MS, Talhouk A, Intermaggio MP*, et al.* Prognostic gene expression signature for high-grade serous ovarian cancer. Ann Oncol 2020, 31(9): 1240-1250.

65. Dangaj D, Bruand M, Grimm AJ, Ronet C, Barras D, Duttagupta PA*, et al.* Cooperation between Constitutive and Inducible Chemokines Enables T Cell Engraftment and Immune Attack in Solid Tumors. Cancer Cell 2019, 35(6): 885-900 e810.

66. Van der Meer JMR, de Jonge P, van der Waart AB, Geerlings AC, Moonen JP, Brummelman J*, et al.* CD34(+) progenitor-derived NK cell and gemcitabine combination therapy increases killing of ovarian cancer cells in NOD/SCID/IL2Rg(null) mice. Oncoimmunology 2021, 10(1): 1981049.

67. Lisco A, Hsu AP, Dimitrova D, Proctor DM, Mace EM, Ye P*, et al.* Treatment of Relapsing HPV Diseases by Restored Function of Natural Killer Cells. N Engl J Med 2021, 385(10): 921-929.

68. Rocha GA, de Melo FF, Cabral M, de Brito BB, da Silva FAF, Queiroz DMM. Interleukin-27 is abrogated in gastric cancer, but highly expressed in other Helicobacter pylori-associated gastroduodenal diseases. Helicobacter 2020, 25(1): e12667.

69. Nagai H, Oniki S, Fujiwara S, Xu M, Mizoguchi I, Yoshimoto T*, et al.* Antitumor activities of interleukin-27 on melanoma. Endocr Metab Immune Disord Drug Targets 2010, 10(1): 41-46.

70. Dibra D, Mitra A, Newman M, Xia X, Cutrera JJ, Gagea M*, et al.* Lack of Immunomodulatory Interleukin-27 Enhances Oncogenic Properties of Mutant p53 In Vivo. Clin Cancer Res 2016, 22(15): 3876-3883.

71. Rizzitelli A, Meuter S, Vega Ramos J, Bird CH, Mintern JD, Mangan MS*, et al.* Serpinb9 (Spi6)-deficient mice are impaired in dendritic cell-mediated antigen cross-presentation. Immunol Cell Biol 2012, 90(9): 841-851.

72. Cooley J, Sontag MK, Accurso FJ, Remold-O'Donnell E. SerpinB1 in cystic fibrosis airway fluids: quantity, molecular form and mechanism of elastase inhibition. Eur Respir J 2011, 37(5): 1083-1090.

73. Radfar S, Wang Y, Khong HT. Activated CD4+ T cells dramatically enhance chemotherapeutic tumor responses in vitro and in vivo. J Immunol 2009, 183(10): 6800-6807.

74. Landmeier S, Altvater B, Pscherer S, Juergens H, Varnholt L, Hansmeier A*, et al.* Activated human gammadelta T cells as stimulators of specific CD8+ T-cell responses to subdominant Epstein Barr virus epitopes: potential for immunotherapy of cancer. J Immunother 2009, 32(3): 310-321.

75. Topalian SL, Hodi FS, Brahmer JR, Gettinger SN, Smith DC, McDermott DF*, et al.* Safety, activity, and immune correlates of anti-PD-1 antibody in cancer. N Engl J Med 2012, 366(26): 2443-2454.

76. Van Allen EM, Miao D, Schilling B, Shukla SA, Blank C, Zimmer L*, et al.* Genomic correlates of response to CTLA-4 blockade in metastatic melanoma. Science 2015, 350(6257): 207-211.

77. Zhang K, Erkan EP, Jamalzadeh S, Dai J, Andersson N, Kaipio K*, et al.* Longitudinal single-cell RNA-seq analysis reveals stress-promoted chemoresistance in metastatic ovarian cancer. Sci Adv 2022, 8(8): eabm1831.

## Supplementary Tables

## Table S1 89 significant genes for OS by univeriate analysis of the blue module.

| **Gene** | **HR** | ***P*** | **lower** | **upper** | **selected by lasso** |
| --- | --- | --- | --- | --- | --- |
| FCGBP | 1.294739174 | 2.75E-05 | 1.147472952 | 1.460905483 | Yes |
| CXCL13 | 0.771778813 | 0.000357706 | 0.669446946 | 0.88975316 | No |
| PDP1 | 1.256844415 | 0.000968368 | 1.097254876 | 1.439645353 | Yes |
| CXCL9 | 0.801568771 | 0.001297992 | 0.700496585 | 0.917224308 | Yes |
| FBXO16 | 0.817069233 | 0.001648627 | 0.720470443 | 0.926619735 | No |
| CCDC34 | 0.815175179 | 0.00201432 | 0.716017574 | 0.928064613 | No |
| HMGN3 | 0.819718389 | 0.002112587 | 0.722131929 | 0.930492352 | No |
| CMBL | 0.808915583 | 0.002143976 | 0.70647578 | 0.926209276 | Yes |
| PIEZO1 | 1.214126075 | 0.002742836 | 1.069359797 | 1.378490318 | No |
| APOL4 | 0.812004446 | 0.002789698 | 0.708391815 | 0.930771933 | No |
| IL27RA | 1.216400663 | 0.005218939 | 1.060178792 | 1.395642493 | Yes |
| CYBRD1 | 1.206555535 | 0.005893622 | 1.055611846 | 1.379082912 | Yes |
| GBP4 | 0.839620497 | 0.005900526 | 0.741378473 | 0.95088083 | No |
| CLEC5A | 1.19289976 | 0.005985496 | 1.051910816 | 1.352785631 | No |
| C1orf131 | 0.836060804 | 0.006001237 | 0.735816586 | 0.949961827 | Yes |
| PSMA6 | 0.824319143 | 0.006276289 | 0.71766722 | 0.946820519 | No |
| VSIG4 | 1.199591671 | 0.006503574 | 1.052228497 | 1.367592856 | Yes |
| CNPY2 | 0.837205098 | 0.006701663 | 0.736289059 | 0.951952725 | No |
| SRP9 | 0.836971953 | 0.007061307 | 0.735325372 | 0.952669495 | No |
| TPGS2 | 0.832914168 | 0.007994077 | 0.727657215 | 0.953396734 | No |
| GCH1 | 0.842455109 | 0.00849336 | 0.741480439 | 0.957180491 | No |
| GBGT1 | 1.181046718 | 0.009313529 | 1.041829488 | 1.33886722 | No |
| CD3D | 0.839953243 | 0.009437835 | 0.736314223 | 0.958179848 | No |
| SELL | 0.842280598 | 0.009790887 | 0.739429375 | 0.959437952 | Yes |
| TSPAN6 | 0.841372995 | 0.010022427 | 0.737725421 | 0.959582654 | Yes |
| GSKIP | 0.845549307 | 0.010418389 | 0.743688539 | 0.961361637 | No |
| C1orf35 | 0.842894111 | 0.010637651 | 0.739297168 | 0.961007985 | No |
| IGSF21 | 1.179142452 | 0.011120306 | 1.038290534 | 1.339101992 | No |
| GTF2F2 | 1.18944693 | 0.01136069 | 1.039940335 | 1.36044728 | Yes |
| STAB1 | 1.180706 | 0.011718587 | 1.037633118 | 1.343506327 | No |
| RASSF2 | 1.180497349 | 0.01227529 | 1.036717104 | 1.344218192 | Yes |
| C2orf74 | 0.852637056 | 0.012578416 | 0.752291554 | 0.96636729 | No |
| CD2 | 0.846489656 | 0.013038566 | 0.742135149 | 0.965517857 | No |
| UBE2L3 | 0.840901609 | 0.014601413 | 0.731725358 | 0.966367377 | Yes |
| NUP35 | 0.849676605 | 0.01462634 | 0.745522267 | 0.968381985 | No |
| COX20 | 0.849601894 | 0.014998627 | 0.745039753 | 0.968838744 | No |
| BTN3A1 | 0.855866736 | 0.015148582 | 0.754849257 | 0.970402849 | Yes |
| PLEKHO2 | 1.178727455 | 0.016334672 | 1.030685893 | 1.348032821 | No |
| RNF32 | 0.849395483 | 0.016739193 | 0.743076006 | 0.970927175 | Yes |
| NXT2 | 0.846147722 | 0.017531641 | 0.737191781 | 0.971207203 | No |
| JADE2 | 1.167005489 | 0.01803589 | 1.026796238 | 1.326360343 | No |
| ADCY9 | 1.165816628 | 0.018336366 | 1.026278113 | 1.324327582 | No |
| PRRT1 | 0.847241287 | 0.019214645 | 0.737464301 | 0.973359384 | No |
| PRKRA | 0.8508176 | 0.021053948 | 0.741699273 | 0.975989347 | No |
| ELP3 | 0.860021565 | 0.0211379 | 0.756544309 | 0.977652047 | Yes |
| DCTN6 | 0.860865982 | 0.021772698 | 0.75744801 | 0.978404101 | No |
| ANAPC13 | 0.866831707 | 0.021951845 | 0.767082847 | 0.979551571 | No |
| PABPN1 | 0.862752824 | 0.022073325 | 0.760311188 | 0.978997081 | Yes |
| RNPEPL1 | 1.162779427 | 0.022568379 | 1.021433315 | 1.323685038 | No |
| TRPM2 | 1.164650812 | 0.022793268 | 1.021442095 | 1.327937747 | No |
| CFI | 0.861000843 | 0.023038481 | 0.756751664 | 0.979611261 | Yes |
| IL2RG | 0.861645616 | 0.023063962 | 0.757789321 | 0.979735591 | Yes |
| PADI2 | 1.158948369 | 0.023349409 | 1.020218018 | 1.316543423 | No |
| FOXP3 | 0.855894594 | 0.023385605 | 0.748161438 | 0.979141023 | No |
| NPAS3 | 0.862393609 | 0.024220611 | 0.758208567 | 0.980894664 | Yes |
| FPR1 | 1.165366546 | 0.025488487 | 1.018944554 | 1.332829329 | No |
| MICB | 0.867418941 | 0.028290241 | 0.763884696 | 0.984985853 | Yes |
| CD3E | 0.864576984 | 0.029651882 | 0.758311734 | 0.985733608 | No |
| TSPAN7 | 0.867071497 | 0.029911433 | 0.762321262 | 0.986215416 | Yes |
| GKAP1 | 0.859023139 | 0.030891044 | 0.748305837 | 0.986121872 | No |
| TTC32 | 0.866072177 | 0.031166285 | 0.759903206 | 0.98707442 | Yes |
| STEAP3 | 1.149391971 | 0.031327286 | 1.012555227 | 1.304720836 | No |
| GIMAP7 | 0.863448615 | 0.032162608 | 0.754922438 | 0.987576304 | Yes |
| KLF16 | 1.151963729 | 0.032917886 | 1.011547605 | 1.31187146 | No |
| NLRC5 | 0.865069964 | 0.033896339 | 0.756641443 | 0.989036551 | No |
| ELP6 | 0.866393445 | 0.034249432 | 0.758671656 | 0.989410368 | No |
| THEMIS2 | 1.153275754 | 0.034720548 | 1.010293481 | 1.316493662 | No |
| FCHSD1 | 1.156105078 | 0.035262407 | 1.010066142 | 1.32325884 | No |
| EPS8 | 1.14692524 | 0.035885063 | 1.009074163 | 1.303608352 | Yes |
| SERPINB9 | 0.863071208 | 0.036335796 | 0.751904298 | 0.99067383 | Yes |
| SIT1 | 0.863598412 | 0.037250312 | 0.752290902 | 0.991374765 | No |
| CSPG5 | 0.861340465 | 0.037431585 | 0.748377692 | 0.991354239 | Yes |
| ME1 | 0.869466608 | 0.037794968 | 0.761957588 | 0.992144699 | Yes |
| GPT2 | 0.867042911 | 0.039115809 | 0.757137533 | 0.992902052 | No |
| SIRPA | 1.147053174 | 0.039445625 | 1.006653243 | 1.307034964 | No |
| DUSP12 | 0.871320018 | 0.039636223 | 0.764173237 | 0.993490137 | No |
| SLC6A12 | 1.14771562 | 0.041478097 | 1.005336322 | 1.310259179 | Yes |
| RIN1 | 1.148681142 | 0.041597065 | 1.005290742 | 1.312524139 | No |
| ABHD14A | 0.873165036 | 0.041900144 | 0.766215557 | 0.995042679 | No |
| ALOX5AP | 1.145254462 | 0.042213167 | 1.004781642 | 1.305365991 | No |
| SUN2 | 1.143399343 | 0.042288313 | 1.004676961 | 1.301276039 | No |
| GNAS | 0.883597407 | 0.042987068 | 0.783799639 | 0.996101987 | Yes |
| CCL8 | 0.870085024 | 0.044131441 | 0.759820609 | 0.996350901 | No |
| ITGAM | 1.142072263 | 0.0453615 | 1.002746747 | 1.300756205 | No |
| JAK2 | 0.868736628 | 0.046812069 | 0.75620231 | 0.998017752 | Yes |
| SERPINB1 | 0.881933704 | 0.047458672 | 0.778903275 | 0.998592614 | Yes |
| CD302 | 1.15141695 | 0.048064082 | 1.001201634 | 1.324169826 | No |
| SPSB1 | 1.134526301 | 0.048501556 | 1.000830932 | 1.286081283 | No |
| SLCO2B1 | 1.139944427 | 0.049970117 | 1.000017087 | 1.299451094 | No |

## Table S2 The FASTA sequences used for *SERPIN1* and *SERPIN9* protein homology modeling.

| **Gene name** | **Protein FASTA sequences** |
| --- | --- |
| SERPIN1 | MEQLSSANTRFALDLFLALSENNPAGNIFISPFSISSAMAMVFLGTRGNTAAQLSKTFHFNTVEEVHSRFQSLNADINKRGASYILKLANRLYGEKTYNFLPEFLVSTQKTYGADLASVDFQHASEDARKTINQWVKGQTEGKIPELLASGMVDNMTKLVLVNAIYFKGNWKDKFMKEATTNAPFRLNKKDRKTVKMMYQKKKFAYGYIEDLKCRVLELPYQGEELSMVILLPDDIEDESTGLKKIEEQLTLEKLHEWTKPENLDFIEVNVSLPRFKLEESYTLNSDLARLGVQDLFNSSKADLSGMSGARDIFISKIVHKSFVEVNEEGTEAAAATAGIATFCMLMPEENFTADHPFLFFIRHNSSGSILFLGRFSSP |
| SERPIN9 | METLSNASGTFAIRLLKILCQDNPSHNVFCSPVSISSALAMVLLGAKGNTATQMAQALSLNTEEDIHRAFQSLLTEVNKAGTQYLLRTANRLFGEKTCQFLSTFKESCLQFYHAELKELSFIRAAEESRKHINTWVSKKTEGKIEELLPGSSIDAETRLVLVNAIYFKGKWNEPFDETYTREMPFKINQEEQRPVQMMYQEATFKLAHVGEVRAQLLELPYARKELSLLVLLPDDGVELSTVEKSLTFEKLTAWTKPDCMKSTEVEVLLPKFKLQEDYDMESVLRHLGIVDAFQQGKADLSAMSAERDLCLSKFVHKSFVEVNEEGTEAAAASSCFVVAECCMESGPRFCADHPFLFFIRHNRANSILFCGRFSSP |

**Table S3 Molecular docking results of the top 15 compounds for *SERPINB1*.**

| **Rank** | **TTD Drug ID** | **Compound name** | **XP GScore** | **2D-structure** |
| --- | --- | --- | --- | --- |
| 1 | D0X7ZC | 9-Aminomethyl-9H-fluorene-3,4-diol | -8.902 | 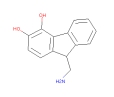 |
| 2 | D0R3JB | Mitoxantrone | -8.855 | 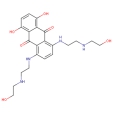 |
| 3 | D08FPM | Dhaq diacetate | -8.855 | 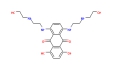 |
| 4 | D0O1EY | 9-Aminomethyl-9H-fluorene-2,5,6-triol | -8.845 | 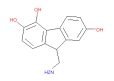 |
| 5 | D0OH5V | PIROXANTRONE | -8.803 | 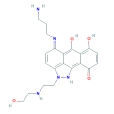 |
| 6 | D04ZLJ | LOSOXANTRONE | -8.684 | 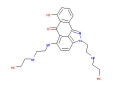 |
| 7 | D0N1WM | A-68930 | -8.397 | 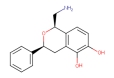 |
| 8 | D09CUJ | 6-fluoro-noradrenaline | -8.199 | 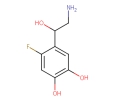 |
| 9 | D05RWZ | 2-fluoronorepinehprine | -8.199 | 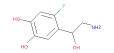 |
| 10 | D0U6IL | 1-Aminomethyl-isochroman-5,6-diol | -8.076 | 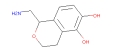 |
| 11 | D0O1DU | D-phenylalanyl-N-(3-chlorobenzyl)-L-prolinamide | -8.000 | 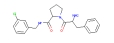 |
| 12 | D04BYL | 6-fluoronorepinehprine | -7.771 | 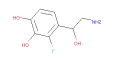 |
| 13 | D0K3JC | D-phenylalanyl-N-(3-methylbenzyl)-L-prolinamide | -7.816 | 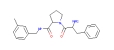 |
| 14 | D0UQ6A | PMID2231594:C3q | -7.613 | 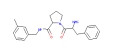 |
| 15 | D05NBK | 1-Aminomethyl-3-cyclohexyl-isochroman-5,6-diol | -7.607 | 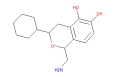 |

## Table S4 Molecular docking results of the top 15 compounds for *SERPINB9*.

| **Rank** | **TTD Drug ID** | **Compound name** | **XP GScore** | **2D-structure** |
| --- | --- | --- | --- | --- |
| 1 | D0X3XE | CGP 40336A | -11.279 | 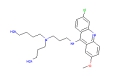 |
| 2 | D0OH5V | PIROXANTRONE | -9.095 | 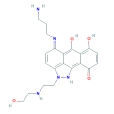 |
| 3 | D04ZLJ | LOSOXANTRONE | -9.070 | 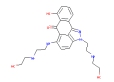 |
| 4 | D0X7ZC | 9-Aminomethyl-9H-fluorene-3,4-diol | -8.768 | 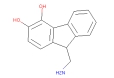 |
| 5 | D04NXR | RO-16-8714 | -8.875 | 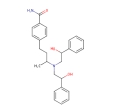 |
| 6 | D0O1EY | 9-Aminomethyl-9H-fluorene-2,5,6-triol | -8.671 | 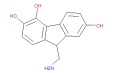 |
| 7 | D0Z1UA | Nebivolol | -8.511 | 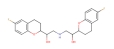 |
| 8 | D0P8AW | Ro-21-7767 | -8.511 | 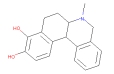 |
| 9 | D0OS2I | US8592455, 3 | -8.387 | 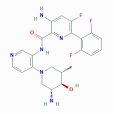 |
| 10 | D03KAQ | 2-Benzylaminomethyl-3-hydroxymorphinan | -8.382 | 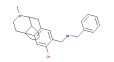 |
| 11 | D0D3VT | Dihydrexidine | -8.138 | 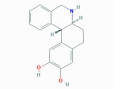 |
| 12 | D0AZ8C | Silymarin | -8.079 | 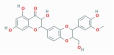 |
| 13 | D0B7CZ | Teloxantrone | -8.111 | 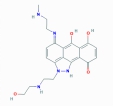 |
| 14 | D09OPS | SN-28049 | -7.970 | 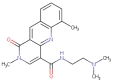 |
| 15 | D0MT1N | N-Ethyl-2-methylnorapomorphine hydrochloride | -7.955 | 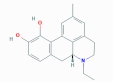 |

## Table S5 Patient information of all datasets in this study.

| **Variables** | **TCGA-OV (Training)** | **Validation 1** | **Validation 2 (Metaarray)** | | |
| --- | --- | --- | --- | --- | --- |
|  |  | **GSE32062** | **GSE17260** | **GSE9891** | **GSE26193** |
| **Total** | 378 | 260 | 110 | 258 | 79 |
| **Age** |  |  |  |  |  |
| <60 | 198 | - | - | 135 | - |
| ≥60 | 180 | - | - | 123 | - |
| **TNM stage** |  |  |  |  |  |
| I/II | 157 | - | - | 33 | 17 |
| III/IV | 79 | 260 | 110 | 224 | 62 |
| **Grade** |  |  |  |  |  |
| G1/G2 | 46 | 131 | 67 | 102 | 23 |
| G3/G4 | 322 | 129 | 43 | 153 | 56 |
| **Lymphatic_invasion** |  |  |  |  |  |
| Yes | 101 | - | - | - | - |
| No | 48 | - | - | - | - |
| **Venous_invasion** |  |  |  |  |  |
| Yes | 64 | - | - | - | - |
| No | 41 | - | - | - | - |
| **Progression** |  |  |  |  |  |
| Yes | 277 | 193 | 76 | 182 | 63 |
| No | 101 | 67 | 34 | 76 | 16 |
| **Tumor burden** |  |  |  |  |  |
| Tumor free | 84 | - | - | - | - |
| With tumor | 247 | - | - | - | - |

## Supplementary Figures and Legends


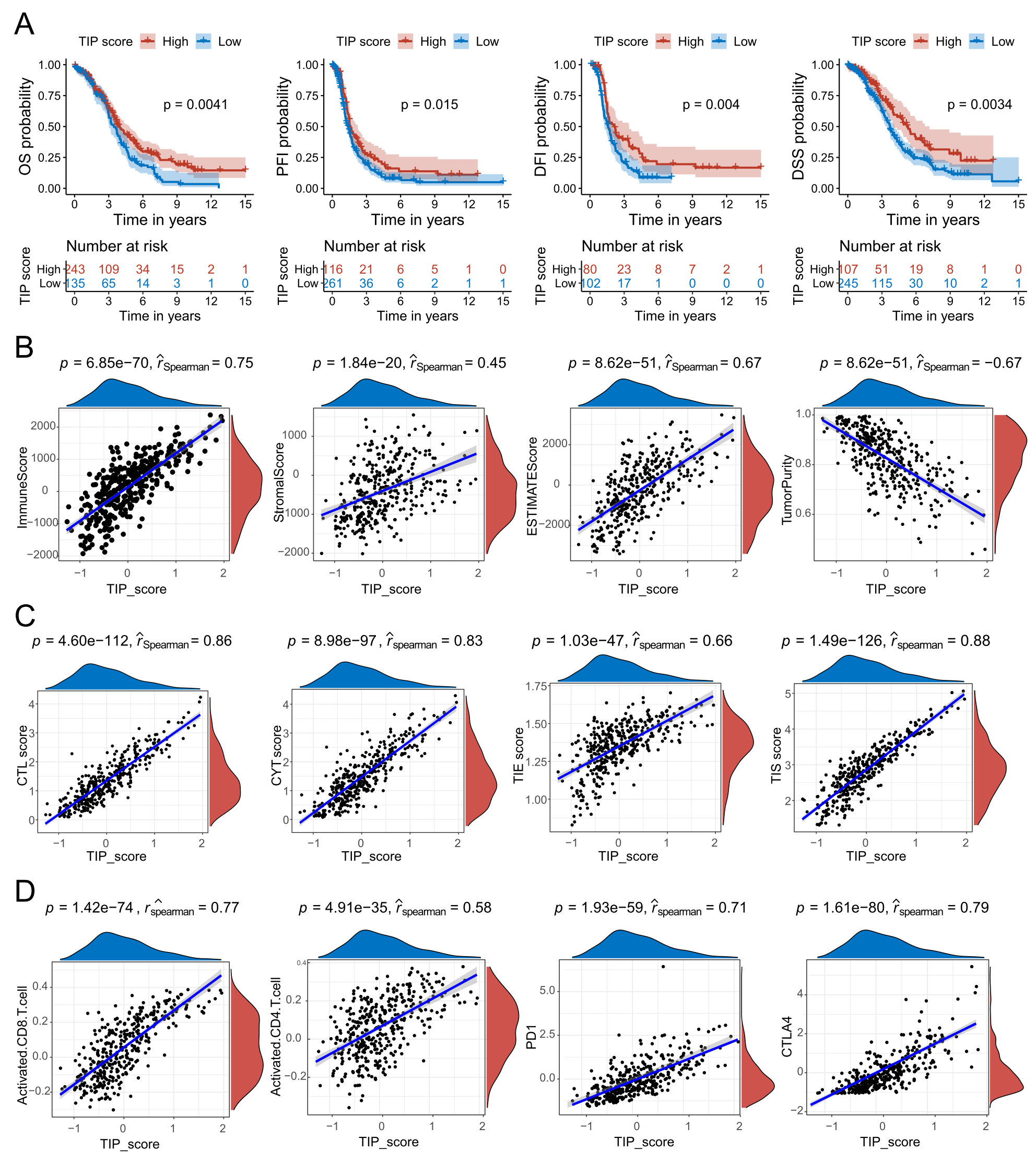


**Figure S1 Correlation of TIP score with OC prognosis and immune-related indicators.** **(A)** Kaplan-Meier survival plots of OC patients with low and high TIP scores for OS, PFI, DFI, and DSS. **(B)** Relationship between TIP score and immune score, stromal score, estimate score, and tumor purity. **(C)** Relationship between TIP score and CTL score, CTY score, TIE score, and TIS score. **(D)** Relationship between TIP score and the infiltration of CD8 and CD4 T cells and the expression of *PD-1* and *CTLA-4*.


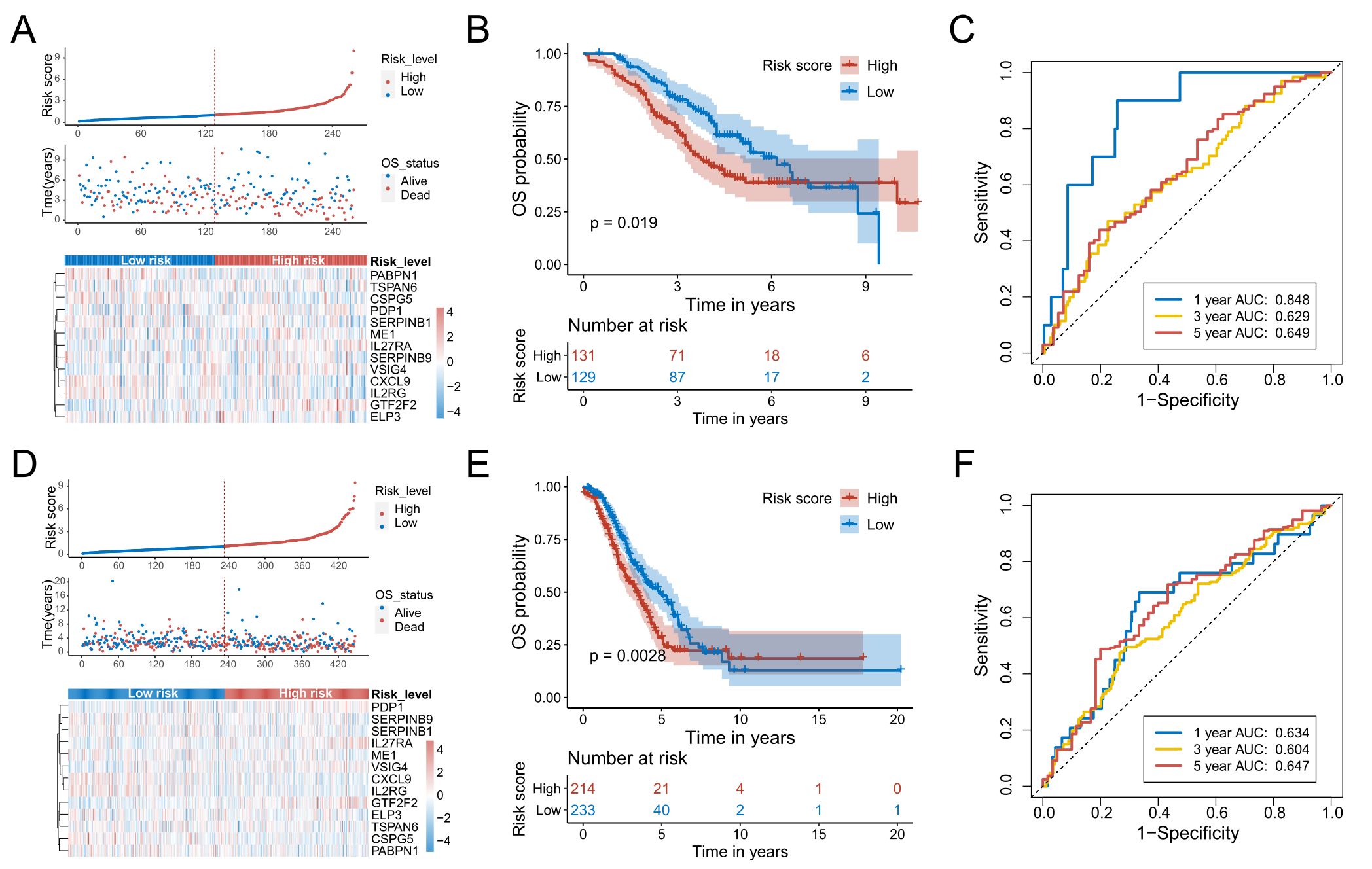


**Figure S2 Assessment of TIPRGPI with the two external validation datasets. (A-C)** Risk stratification of OC patients, Kaplan-Meier survival plots, and tROC curves of validation dataset 1 (GSE32062). **(D-F)** Risk stratification of OC patients, Kaplan-Meier survival plots, and tROC curves of validation dataset 2 (a meta-array dataset combining GSE17260, GSE9891, and GSE26193).


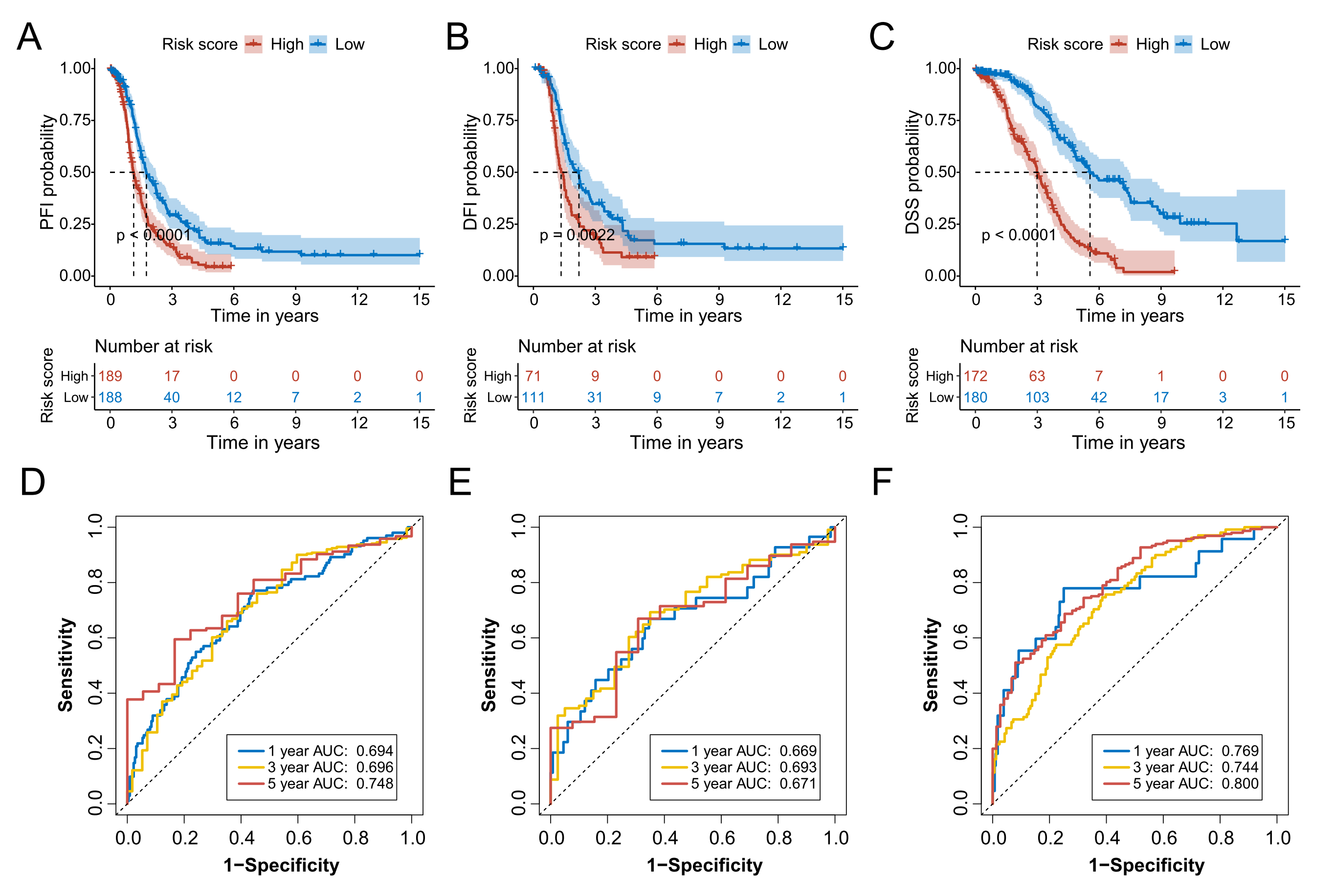


**Figure S3 TIPRGPI could predict the PFI, DFI, and DSS of OC patients. (A-C)** Kaplan-Meier curves of TIPRGPI risk score for the PFI, DFI, and DSS of OC patients. **(D-F)** Time dependent ROC plots of TIPRGPI risk score to evaluate the predictive accuracies of PFI, DFI, and DSS.


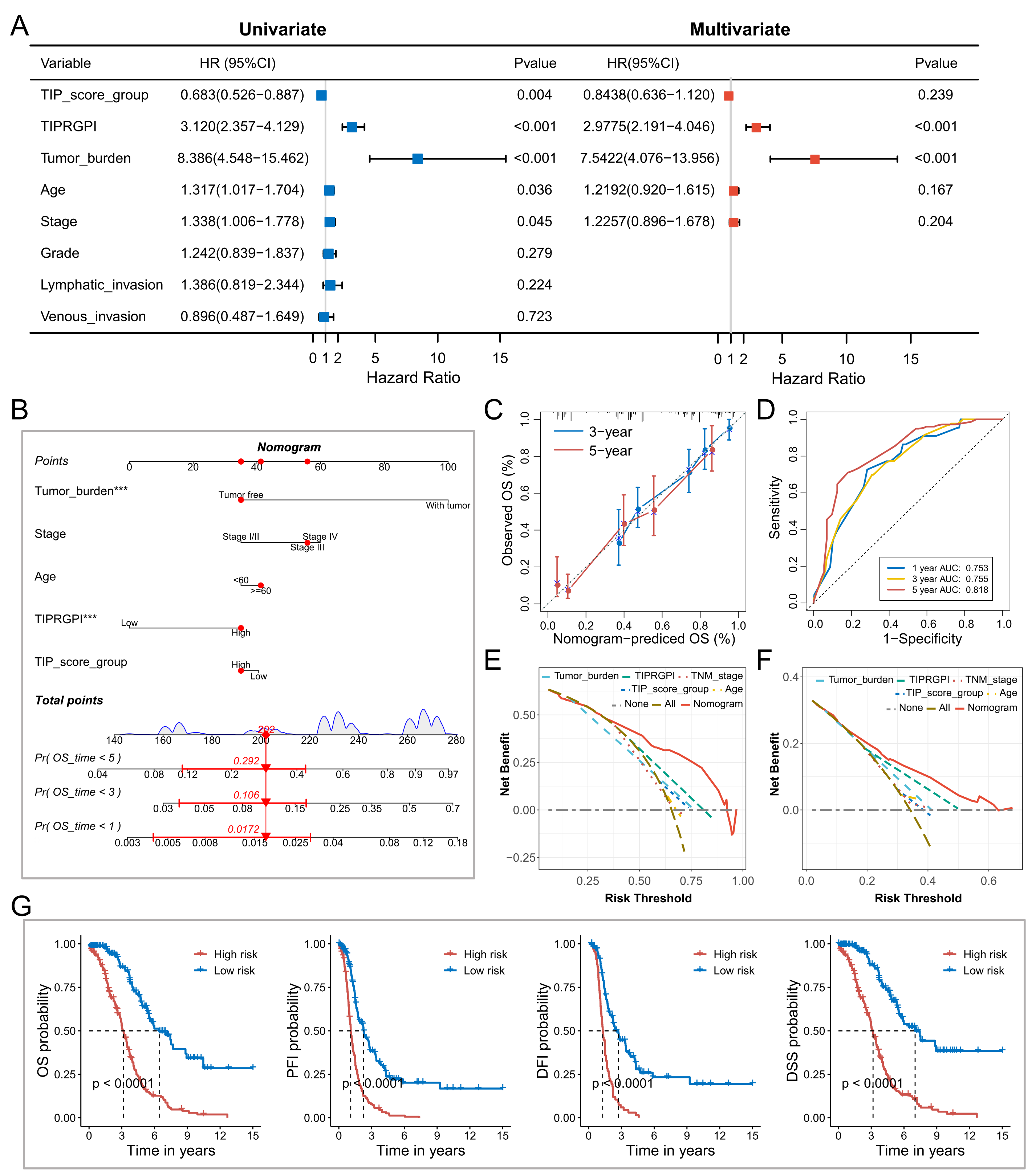


**Figure S4 Evaluation of clinicopathologic features and the TIPRGPI-integrated nomogram construction. (A)** Univariate and multivariate analysis of TIPRGPI and clinicopathologic factors. **(B)** Nomogram for predicting OS in the TCGA-OV dataset. **(C)** Calibration curves showing the consistency between the nomogram predicted and the actual 3-year and 5-year OS. **(D)** tROC curve analysis for the nomogram in 1-year, 3-year, and 5-year survival. **(E, F)** Decision curves showing the comparison of net benefits of tumor burden, TIPRGPI, TNM stage, TIP score, age, and the nomogram for 3-year **(E)** and 5-year **(F)** OS. **(G)** K-M survival analysis of the nomogram for OS, PFI, DFI, and DSS.


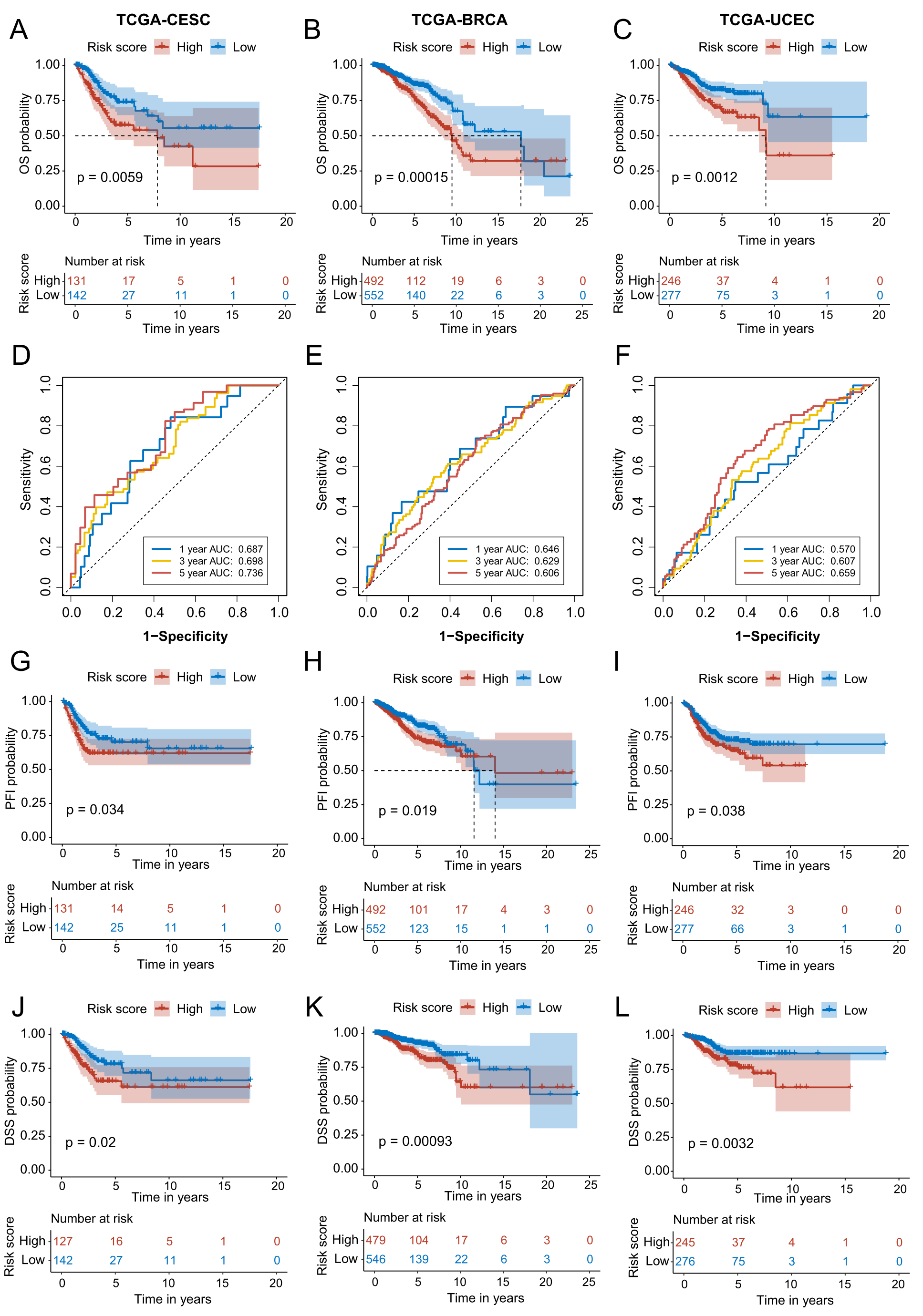


**Figure S5 TIPRGPI had the potential to predict the prognosis of other gynecological tumors.** **(A-C)** K-M survival analysis of the TIPRGPI low- and high-risk groups from TCGA-CESC (*n* = 273), TCGA-BRCA (*n* = 1044), and TCGA-UCEC (*n* = 523) sets for OS. **(D-F)** tROC curve analysis for the three datasets. **(G-L)** K-M survival analysis for PFI **(G-I)** and DSS **(J-L)** in the three datasets.


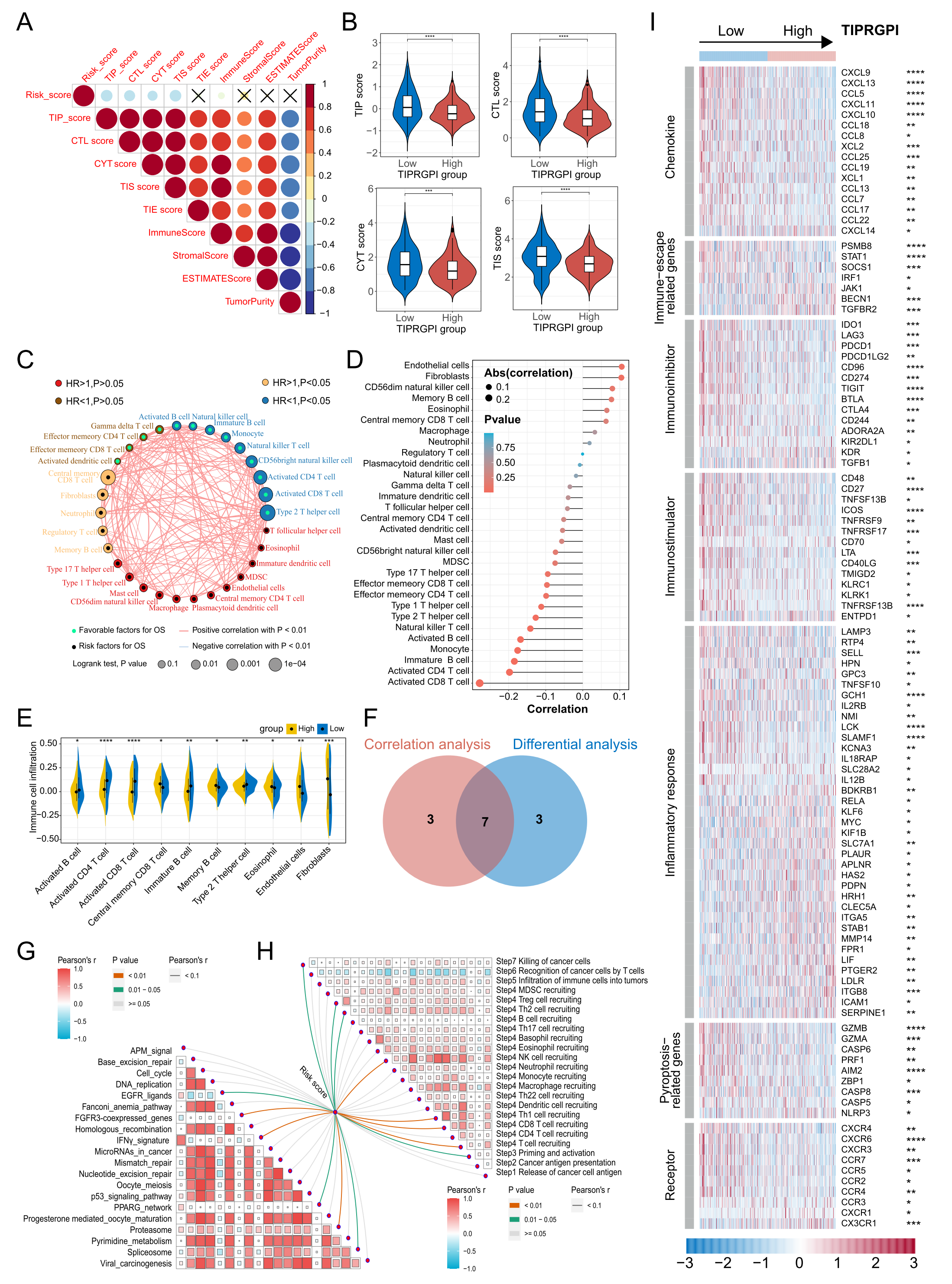


**Figure S6 TIPRGPI was correlated with OC immune status. (A)** Significant correlations between TIPRGPI and TIP score (*P* < 0.001), CTL score (*P* < 0.001), CYT score (*P* < 0.001), TIS score (*P* < 0.001), and immune score (*P* = 0.047). **(B)** Differential distributions of TIP score, CTL score, CYT score, TIS score between distinct TIPRGPI risk groups. **(C)** Interactive network of 30 TIME cell types with prognostic significance. The size of each cell indicates the survival influence assessed by log-rank tests. **(D)** Correlation analysis of immune infiltration and TIPRGPI. **(E)** Significantly differential immune cell infiltration between the TIPRGPI low- and high-risk groups. **(F)** Venn diagram showing the intersected TIME cells by correlation analysis and differential analysis. **(G)** Correlations of TIPRGPI and the enrichment scores of multiple therapeutic-related signatures. **(H)** Correlations of TIPRGPI and the activity of each step in the cancer-immunity cycle. **(I)** Variations in mRNA expression of immune-related genes including immunomodulators, inflammatory response-related genes, immune escape-related genes, and pyroptosis-related genes. **P* < 0.05, ***P* < 0.01, ****P* < 0.001, and *****P* < 0.0001.


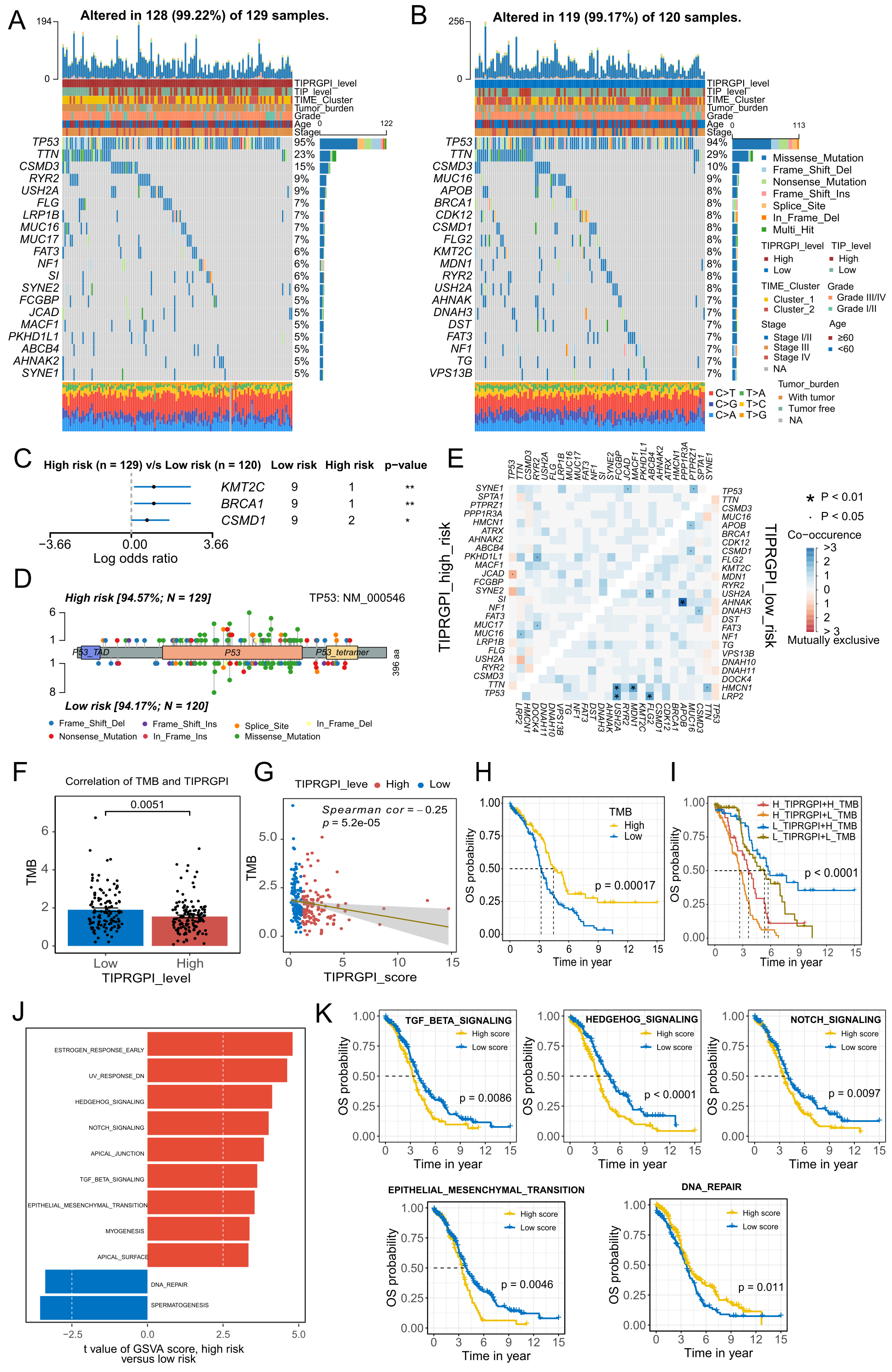


**Figure S7 Mutation patterns and signaling pathway analysis regarding TIPRGPI.** **(A, B)** Waterfall plots of the TIPRGPI low- **(A)** and high-risk group **(B)**. **(C)** Forest plots showing the most differentially mutated genes between TIPRGPI low-risk and high-risk groups. **(D)** Lollipop plot of mutation sites of *TP53*. **(E)** Co-occurrence and exclusivity of mutations of the top 25 mutated genes. **(F)** Significant TMB difference determined by Wilcoxon test in the TIPRGPI low-risk and high-risk groups. **(G)** Scatterplots depicting the negative correlation (Spearman correlation = -0.25, *P* = 5.2e-05) between TIPRGPI risk scores and mutation load in the TCGA-OV dataset. The correlation between ICI scores and mutation load is shown (*P* = 0.0058). **(H)** Kaplan-Meier curves for the high- and low-TMB groups of the TCGA-OV dataset. **(I)** Kaplan-Meier survival curves for OC patients of the TCGA-OV dataset stratified by TMB and TIPRGPI. **(J)** Differential hallmark pathways between the TIPRGPI low-risk and high-risk groups. **(K)** Kaplan-Meier survival analyses of well-established oncogenic pathways for OS. **P* < 0.05, ***P* < 0.01.


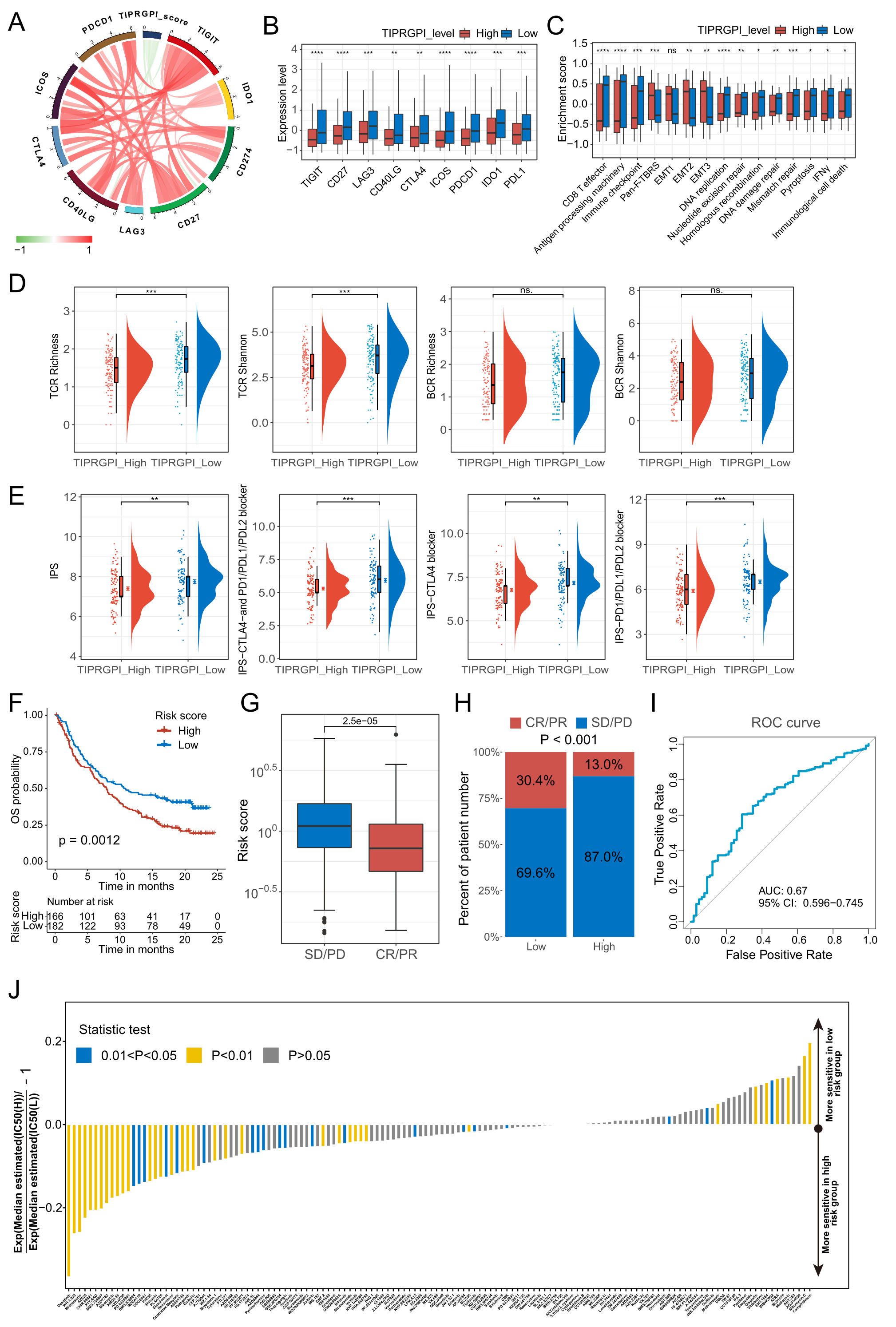


**Figure S8 Evaluation of TIPRGPI susceptibility prediction for therapeutic responses in OC. (A)** Chord diagrams presenting the correlation between the expression of multiple immune checkpoints and TIPRGPI risk score. **(B)** Differential expression levels of multiple immune checkpoints in the TIPRGPI low- and high-risk group. **(C)** Enrichment scores of immune-related signatures in the two risk groups. **(D)** Comparisons of TCR / BCR repertoire indexes the TIPRGPI low- and high-risk group by Wilcoxon tests. **(E)** Comparisons of IPS scores between the TIPRGPI low- and high-risk groups by Wilcoxon tests. **(F)** Kaplan-Meier survival curve indicating the prognostic value of TIPRGPI in the IMvigor210 cohort. **(G)** Differential risk scores between the SD/PD and CR/PR group. **(H)** Barplots demonstrating a higher percentage of CR/PR patients in the low-risk group. **(I)** ROC curves illustrating the good discriminative capacity of TIPRGPI for therapeutic responses. **(J)** Ratio of normalized IC50 values for 138 chemo-/targeted therapy drugs in the TIPRGPI-high and low risk groups of OC patients. ***P* < 0.01, ****P* < 0.001, *****P* < 0.0001, *ns*, not significant.


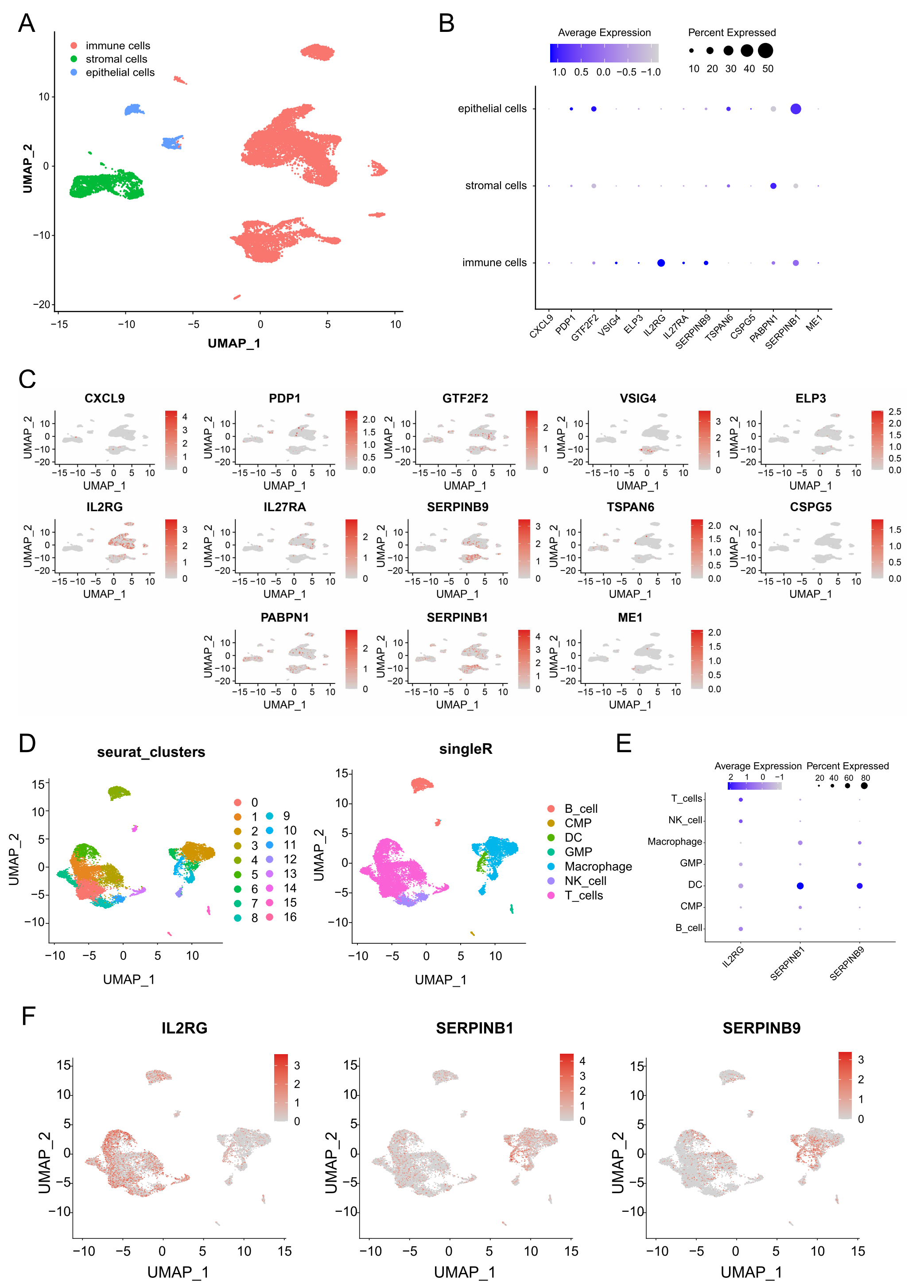


**Figure S9 Single-cell RNA sequencing analysis of TIPRGPI genes. (A)** Clusters identification of immune cells, stromal cells, and epithelial cells. **(B)** TIPRGPI gene expression distributions in different cell types. **(C)** UMAP plots of TIPRGPI model genes in all clusters. **(D)** UMAP plot of SingleR annotated immune cell types. **(E)** Dot plot of the expression levels of *IL2RG*, *SERPINB1*, and *SERPINB9* in different immune cell types. **(F)** UMAP plots of *IL2RG*, *SERPINB1*, and *SERPINB9* in clusters of immune cells.


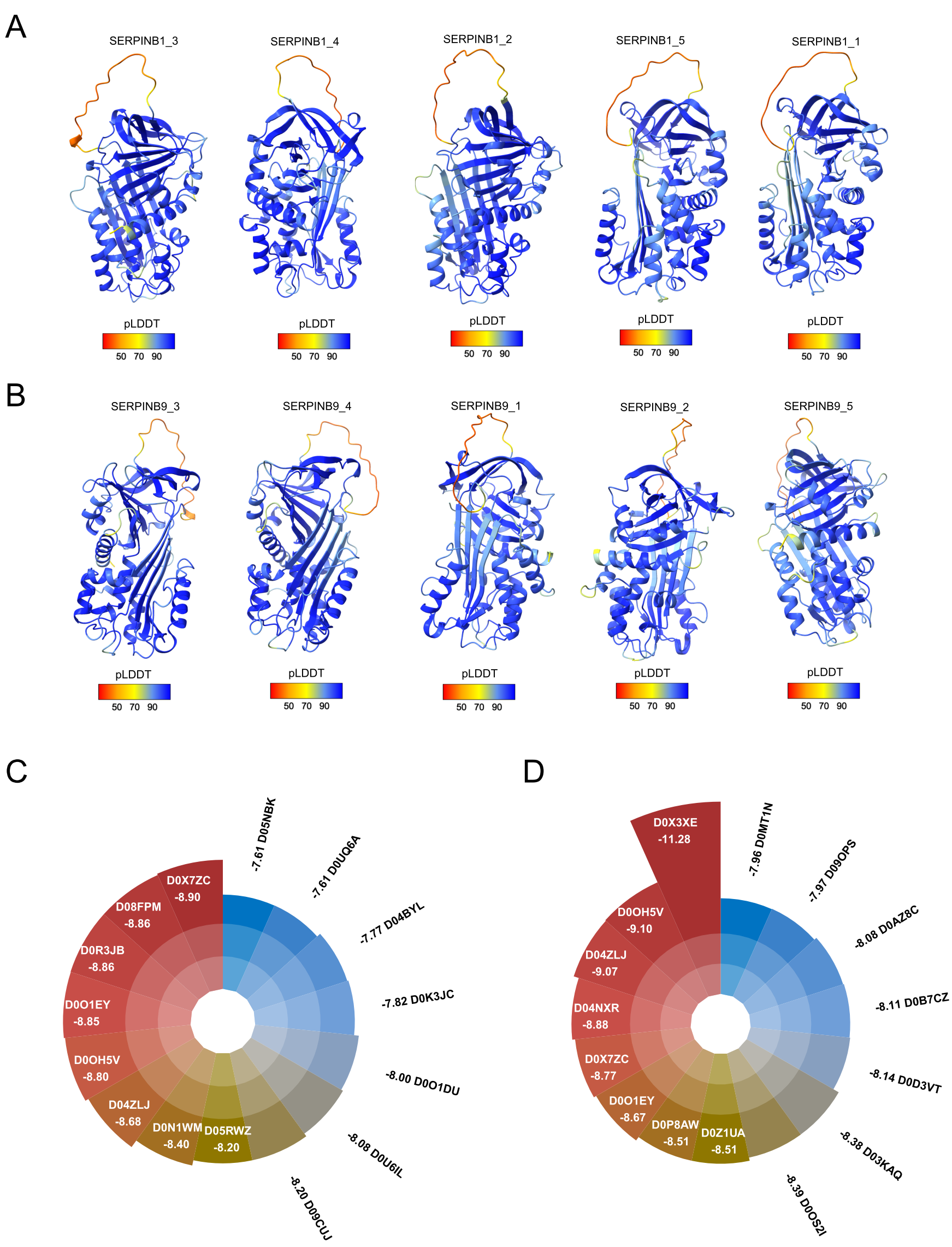


**Figure S10 *SERPINB1* and *SERPINB9* structure predictions and small molecules virtual docking. (A, B)** Five AlphaFold2 predicting relaxed models for *SERPINB1* **(A)** and *SERPINB9* **(B)**, respectively. **(C, D)** Top 15 drugs with the highest glide scores for *SERPINB1* **(C)** and *SERPINB9* **(D)**, respectively.
